# Contemporary in vivo rodent electroconvulsive therapy (ECT) models in translational depression research: a systematic review

**DOI:** 10.1038/s41398-025-03749-x

**Published:** 2025-11-29

**Authors:** Evangelos Kokolakis, Michael G. Gottschalk, Sarah Kläffgen, Jan M. Deussing, Angelika Erhardt, Julius C. Pape, Iven-Alex von Mücke-Heim

**Affiliations:** 1 Max Planck Institute of Psychiatry, Research Clinic and Outpatient Department, Kraepelinstraße 2-10, 80804 Munich, Germany; 2 Max Planck Institute of Psychiatry, Molecular Neurogenetics, Kraepelinstraße 2-10, 80804 Munich, Germany; 3 Max Planck Institute of Psychiatry, Department Genes and Environment, Kraepelinstraße 2-10, 80804 Munich, Germany; 4 University of Würzburg, Department of Psychiatry, Clinical Anxiety Research, Josef-Schneider-Straße 2, 97080 Würzburg, Germany; 5 Cornell University, Department of Psychiatry, Weill Cornell Medicine, New York, NY USA

**Keywords:** Molecular neuroscience, Pathogenesis, Biomarkers

## Abstract

Electroconvulsive therapy (ECT) has been established as an efficacious and safe treatment for severe and/or treatment-resistant depression. However, despite decades of research, the exact biological signature of the mechanism of action of ECT has yet to be elucidated. As a translational tool, electroconvulsive stimulation (ECS), the preclinical rodent equivalent of ECT, offers the unique opportunity for further knowledge under controlled laboratory conditions. Here, for the first time, a systematic review following the PRISMA 2020 statement is presented, covering mouse and rat studies investigating the biobehavioral effects of ECS in chronic stress-based depression models. For this purpose, the PubMed and Web of Science databases (period: 01.01.2000 to 05.10.2023) were screened for different key word combinations (search terms: depression, chronic stress, electroconvulsive shock, rats, mice). The search yielded a total of 1455 records. After filtering, a total of 48 studies were included in this review (n = 7 mice, n = 41 rats). Across the reviewed studies, the most common experimental conditions were 4 weeks of chronic unpredictable mild stress (CUMS) in adult male rats treated with bilateral ear clip ECS for 1 week (parameters: bidirectional square wave, 1.5 ms pulse width with 800 mA at 125 Hz, 1.2 sec stimulation duration, 120 mC charge) using no, propofol, or isoflurane anesthesia. The outcome measures were centered around anhedonia-related behaviors and hippocampal protein levels. Summary odds across different behavioral domains revealed antidepressive effects of ECS on anhedonia (14.5), locomotion (6.0), despair (4.5), and anxiety (2.0), accompanied by memory impairments (0.1). Risk of bias assessment suggested considerable risk, primarily due to unreported information on missing data and blinding. Based on our analysis of the evidence, methodological suggestions for future studies were developed. This review will help to further unlock the translational potential of the ECS to generate much needed insights into the molecular correlates of ECT, with special regard to treatment response and prognosis for depression patients.

## Introduction

Depressive disorders are among the most burdensome disorders known in the healthcare sector [1]. Approximately one in five people will experience a depressive illness once in their life, and current global trend analyses on prevalence and incidence rates suggest a steady increase [2, 3]. Although our understanding of the aetiopathological mechanisms of depression has drastically increased in recent years and novel biological treatments are emerging [4, 5], clinical outcomes still remain unsatisfactory. On average, 40–50% of depression patients experience a recurring disease course with increasing prior episode frequency, while up to 25% convert to a chronic type [6–8]. Moreover, suicide risk is elevated in depressed patients compared to that in the general public by up to 20-fold [9], and a long-term cumulative incidence of suicide of ♀:♂ = 3.8%:6.7% in depressed individuals vs. ♀:♂ = 0.26%:0.72% in healthy individuals has been reported [10]. In addition, there is a considerable incidence skew to the disadvantage of female sex (2-fold) and a low socioeconomic status (3-fold) [11, 12]. In addition to psychotherapeutic strategies and pharmacotherapy [13], neurostimulation methods are among the more effective treatment options [14]. The most prominent and impactful therapy is electroconvulsive therapy (ECT) [15, 16]. Despite the safety and high effectiveness of ECT (e.g., a clinical effect size up to 3 times that of typical antidepressants) and its antisuicidal effects [17–22], which biomolecular conditions determine ECT success at the individual patient level has not been determined [17, 23, 24]. Although lacking ultimate empirical clarity, the existing body of evidence suggests that ECT has pro-neuroplastic effects on mood disorders primarily via normalization of brain-derived neurotrophic factor (BDNF) levels, likely mediated by immune mechanisms, eventually normalizing connectivity and networks in the brain [24, 25]. To improve the precision and long-term outcomes of ECT and to provide patients with a biologically driven risk-benefit assessment prior to and during treatment, valid biomarkers and prognostic models are needed [17]. The latter could augment established clinical prognostic markers such as episode duration, depression severity, or age [26]. To achieve this goal, qualitatively improved translational studies are imperative.

Despite rapid progress in noninvasive and in vitro research methodologies for studying patients with mental disorders over the last two decades, significant limitations remain with regard to the capacity to both disentangle and mimic in vivo brain structure and functions. Moreover, ethical and practical boundaries limit the study of cerebral structure and function in the living human brain [27, 28]. Arguably, preclinical models of mental disorders have advantages and drawbacks that vary according to the neuropsychiatric disorder under investigation and research aim [27, 29, 30]; however, they remain vital research tools in translational neuroscience and neuropsychiatry for the time being [31, 32]. For ECT, the rodent model counterpart is termed electroconvulsive shock (ECS) [33]. Chronic stress-based preclinical models are the most common models for studying the effect of ECS in depressive conditions. In general, rodent depression models aim to mimic the complex aetiopathology of depressive disorders by applying early life adversity, stress in adulthood or biological interventions either alone or in combination [34]. For ECS, an electrical stimulus is administered via either implanted, corneal or ear electrodes to induce generalized tonic‒clonic seizures [35], while electrical stimulation, electrode placement, and other parameters, including but not limited to sex, strain, and depression model vary significantly among studies. These technical and methodological incongruences introduce significant between-study heterogeneity. The latter is apparent in the literature and reduces validity and limits transferability and complicates the generalizability of findings. To improve these circumstances, we believe that a systematic and critical analysis of the current body of evidence can support our understanding of inherent and modifiable factors associated with the advantages and drawbacks of ECS in rodent models of depression.

For this purpose, available preclinical rodent studies applying ECS to model ECT effects in depressive conditions will be expanded upon in the form of a systematic literature review. Available preclinical evidence will first be systematically analyzed and then discussed, focusing on translational potential and validity. The generated insight could help advance valid between-species translation of ECT in depression treatment and inform both clinical and preclinical study designs in the future. Since systematic preclinical reviews are still in their infancy [36], it is not surprising that this is, to the best of our knowledge, the first ever systematic review focusing exclusively on the use of ECS as the preclinical equivalent of ECT in rodent depression models.

## Methods

To identify, select, report, and interpret proper studies within the available body of evidence, we used the Preferred Reporting Items for Systematic Reviews and Meta-Analyses statement in its current version (PRISMA 2020) and its corresponding checklist as methodological guidelines [37]. However, since the PRISMA 2020 framework was designed to evaluate the effects of health care interventions, it is only partially useful for obtaining preclinical evidence. For this reason, we have adapted the suggested checklist items for this review and provided arguments for our changes, wherever appropriate. In addition, we have provided detailed table of the reviewed studies in the supplement modeled after and informed by the ARRIVE guidelines’ essential 10 [38, 39].

### Search strategy

To identify suitable publications, the National Library of Medicine’s PubMed database, which includes MEDLINE, as well as Clarivate’s Web of Science Core Collection (WoS), were searched using the following search terms: (i) (depression) AND (electroconvulsive shock) AND (mice); (ii) (chronic stress) AND (electroconvulsive shock) AND (mice); (iii) (depression) AND (electroconvulsive shock) AND (rats); (iv) (chronic stress) AND (electroconvulsive shock) AND (rats); (v) (depression) AND (electroconvulsive stimulation) AND (mice); (vi) (chronic stress) AND (electroconvulsive stimulation) AND (mice); (vii) (depression) AND (electroconvulsive stimulation) AND (rats); (viii) (chronic stress) AND (electroconvulsive stimulation) AND (rats).To maximize the database search yield, search terms were run exclusively on the “All fields” function of PubMed and Web of Science, and no records including doublets were removed prior to screening. In addition, works linked to the individual publications generated from the search terms displayed on the PubMed website in the “Similar articles” and “Cited by” sections were screened to identify further relevant publications. The review was not registered.

### Selection criteria

All original preclinical or translational research published in peer-reviewed journals between 01.01.2000 and 5 October 2023 was considered. The inclusion criteria for review were (i) the use of recognized and aetiopathologically plausible rodent models of clinical depression within the scientific community in combination with (ii) electroconvulsive shock (ECS) as a proxy for ECT to (iii) study beneficial and/or adverse biobehavioral effects in vivo using both biological readouts and behavioral phenotyping. The rationale for including only studies that used both behavioral and biological readouts was to increase the translatability and comparability of the findings with those of clinical trials. The focus on more recent studies was chosen, in order to reflect current experimental practices, methodologies and reporting standards along with contemporary benchmarks in animal welfare.

Although no single model organism or paradigm can yet actually mimic the complex nature of the gene‒environment interaction aetiopathology of clinical depression, chronic stress exposure—with or without genetic vulnerability—is considered most appropriate for reflecting its neuropsychiatric disease complexity at large [31, 34]. Accordingly, we included all models using either a single or a combination of chronic biological (e.g., repeated LPS injection, selective breeding or genetic manipulation), early life (e.g., maternal separation or limited nesting and bedding) or adult psychosocial and physical stressors (e.g., chronic restraint or isolation, repeated social defeat, unpredictable chronic stress) as well as mixed paradigms (e.g., learned helplessness paradigms). We considered ≥ 7 days of stress exposure as the threshold for chronic stress exposure [32]. For genetic and surgical models, this time criterion was not applicable since they employ a biological causation that permanently changes stress vulnerability and thus results in stress. For a detailed review of common rodent depression models, we refer interested readers to Planchez et al. 2019 [40]. The exclusion criteria were (i) abstracts without full-text publication, conference papers, reviews, meta-analyses, commentaries, letters, perspectives, preprints, nonpeer reviewed journals, or retracted publications; (ii) studies using acute or only subchronic stressor application (timer criterion for chronic stress: ≥ 7 days); (iii) ECS in disorder models or experimental conditions not related proximately to depression; (iv) studies using ECS alone or with only biological and no relevant behavioral readouts in a depression model; and (v) studies using ECS in chronic stress-based rodent models with behavioral but no biological measurements.

### Selection process

The database search was independently conducted by two researchers (EK, SK). For this purpose, publication titles and abstracts were screened for the aforementioned search terms (i) to (viii). Promising records were subsequently retrieved and collated with the predefined inclusion and exclusion criteria of this review. Preliminary extracted data were subsequently compared between EK and SK and, in case of discrepancies, discussed with the IvMH. Throughout the selection and extraction process, senior researchers (IvMH, JCP) oversaw the process and were consulted in case of uncertainty. Final decisions on manuscript inclusion were made jointly by IvMH and JCP.

### Data extraction

Data extraction from identified records was performed by EK, SK, MGG, and IvMH. IvMH oversaw the data extraction process. For each study included, the following parameters were extracted using a pretested and custom-designed form: (a) rodent depression model and animal characteristics: stress paradigm with individual stressors, stress application, duration, species and strain, sex and age; (b) ECS application: application and timing, behavioral assessments; (c) main biobehavioral results focusing on the behaviors evoked by the ECS in stress; and (d) the respective reference (Suppl. Table 1). Bias reduction reporting and comprehensive quality assessment are uncommon in preclinical studies and mostly involve experimenter and analyst blinding, experimental design, including randomization, and missing data. However, due to the unfortunate yet common publishing practice of preclinical studies, that is, the frequent absence of detailed reporting, comprehensive bias assessment was rendered impossible. This issue was aggravated by the fact that available risk of bias tools are tailored to clinical studies and interventions and therefore do not sufficiently map into preclinical designs [41]. Nonetheless, information on measures taken concerning randomization, experimenter blinding, analyst blinding, and missing data was compiled to assess the risk of bias. These four domains were displayed using the risk of bias visualization tool (robvis) and the generic template, which was subsequently individualized [41]. All four domains were weighted equally (low = 1, moderate = 2, high =3; no information = 2) and averaged for the overall risk estimation. The final results were rounded and converted into string variables (e.g., 2.5 average of all bias > high overall risk of bias). If studies lacked information on all risk of bias domains, an overall high risk of bias was assigned.

All biobehavioral results were considered eligible; however, for the purpose of synopsis, only the main summary statistical findings between groups are reported in this review. The latter can be broadly divided into behavioral assessment, metabolite and receptor quantification and/or expression, and immunohistochemistry. The main results were defined based upon the reporting style, i.e., the wording and priorities assigned in the abstract, results, discussion, and conclusion section in the respective study, as well as on consensus within this review’s author group. If uncertainties occurred with regard to the study findings, the corresponding authors were contacted by the IvMH or JCP. However, this did not occur during manuscript preparation. Since preclinical research is by definition highly experimental and thus heterogeneous [42], the authors decided against certainty assessment of individual study results. Nonetheless, overall confidence in the body of evidence was evaluated qualitatively at the bulk level.

### Effect measures

Since preclinical rodent studies, particularly older ones, frequently lack reporting on effect size measures such as Cohen’s D, no traditional effect size measures are reported. Instead, we reported statistical significance levels (p values) and sample sizes for the main biobehavioral findings for descriptive purposes. In addition, we calculated the ratio (summary odds) for beneficial outcomes at the group level, that is, between studies reporting beneficial (e.g., increase in sucrose preference in the sucrose preference test [SPT] or improved recollection and orientation in the Morris water maze [MWM]) and those reporting nonbeneficial (i.e., no significant difference between ECS and controls groups or aggravated stress-associated phenomena in ECS vs. control groups) effects on stress-based phenomena for each behavioral test. For biological results, no such assessment was possible due to the variability of molecular targets. Odds were exclusively calculated for domains in which at least ≥ 5 of the reviewed studies used respective behavioral tests since smaller total sample sizes per assessment would render odds questionable due to multiple sources of bias. Behavioral tests assessing the same domain with merely different terminology or only slightly different specific read-outs were subsumed into one domain odds.

### Synthesis method

Eligible records were summarized to allow easy overview and comparison of the findings. The synthesis method for the Results section follows the logic of data extraction. The records are summarized based on (i) species, strain, age, and sex; (ii) stress paradigm and duration (in days or weeks); (iii) ECS application details and behavioral tests; and (iv) main biobehavioral results (focus: behavioral changes). Heterogeneity, that is for the present review the difference in directionalities of significant findings between studies or no significant findings at all, was assessed qualitatively. No sensitivity analyses were performed.

## Results

**Fig. 1:**
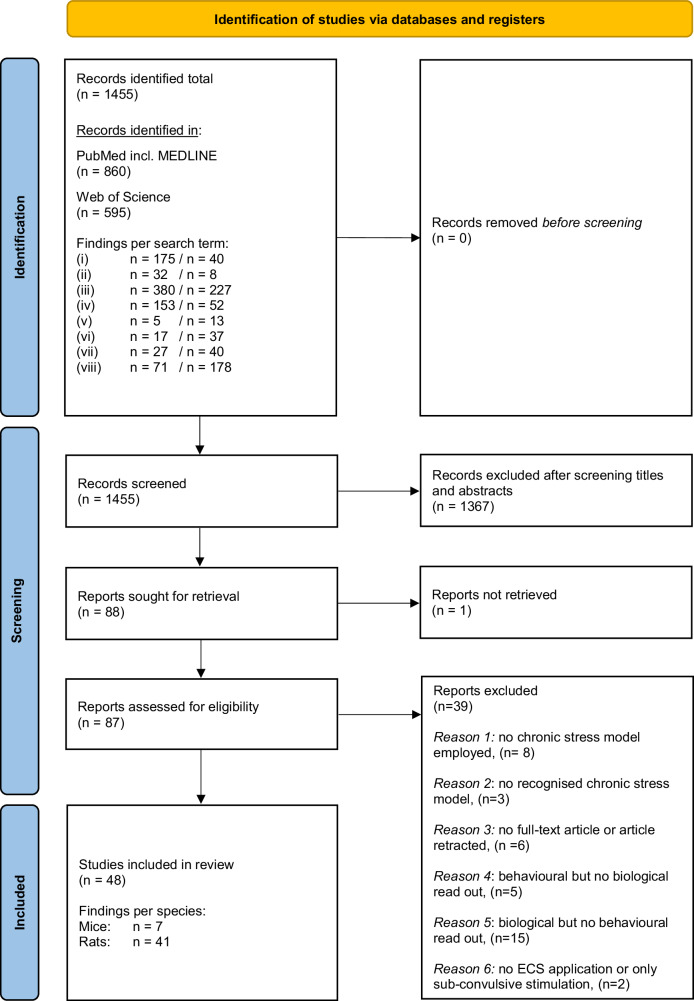
PRISMA 2020 flow chart. For detailed information on the search terms and exclusion criteria, refer to the methods section.

We identified a total of 1455 records published in the National Library of Medicine’s PubMed database between 01.01.2000 and 05.10.2023 (Fig. 1). No filters were applied; therefore, no records were removed prior to screening. The eight search term combinations yielded the following outcomes: (i) (depression) AND (electroconvulsive shock) AND (mice), n = 215; (ii) (chronic stress) AND (electroconvulsive shock) AND (mice), n = 40; (iii) (depression) AND (electroconvulsive shock) AND (rats), n = 607; (iv) (chronic stress) AND (electroconvulsive shock) AND (rats), n = 205; (v) (depression) AND (electroconvulsive stimulation) AND (mice), n = 18; (vi) (chronic stress) AND (electroconvulsive stimulation) AND (mice), n = 54; (vii) (depression) AND (electroconvulsive stimulation) AND (rats), n = 67; (viii) (chronic stress) AND (electroconvulsive stimulation) AND (rats), n = 249. After screening the titles and abstracts, we excluded n = 1367 and sought to retrieve 88 publications. Because n = 1 publication could not be retrieved, we assessed 87 full texts for eligibility. Next, 39 records were excluded because they did not meet the inclusion criteria. Thus, a total of 48 studies were ultimately included. The study characteristics of both the mice and rats are summarized in Table 1. For a detailed overview of stress application, ECS parameters and biobehavioral outcomes including study design and statistics for all reviewed studies, please refer to Suppl. Table 1.

**Table 1:** Characteristics of the reviewed studies using the ECS in chronic stress-based rodent depression models.

	Stress paradigm	Strain, sex, age	Behavioral assessment	Molecular assessment	Ref
1	Chronic unpredictable mild stress (CUMS)	Sprague‒Dawley, male, 2–3 months	Sucrose preference test (SPT), Morris water maze (MWM)	Hippocampal long-term potentiation (LTP) and protein expression	[93]
2	CUMS	Sprague‒Dawley, male, 2–3 months	SPT, MWM	Hippocampal LTP	[94]
3	CUMS	Sprague‒Dawley, male, 2–3 months	SPT, MWM	Hippocampal long-term potentiation (LTP) and protein expression	[95]
4	CUMS	Sprague‒Dawley, male, 2–3 months	SPT, MWM, open field test (OFT)	Hippocampal protein expression	[96]
5	CUMS	Sprague‒Dawley, male, 2–3 months	SPT, MWM	Hippocampal protein expression	[97]
6	CUMS	Sprague‒Dawley, male, 2–3 months	SPT, MWM	Hippocampal mRNA and protein expression	[98]
7	CUMS	Sprague‒Dawley, male, 2–3 months	SPT, MWM	Hippocampal LTP	[99]
8	CUMS	Sprague‒Dawley, male, 2–3 months	SPT, MWM, OFT	Hippocampal protein expression	[100]
9	CUMS	Sprague‒Dawley, male, 2–3 months	SPT, MWM, OFT	Hippocampal protein expression	[101]
10	CUMS	Sprague‒Dawley, male, (specific age not reported)	SPT, MWM, OFT	Hippocampal protein expression	[102]
11	CUMS	Sprague‒Dawley, male, 6–8 weeks	SPT, MWM	Hippocampal protein expression	[103]
12	CUMS	Sprague‒Dawley, male, adult (specific age not reported)	MWM, OFT	Hippocampal glutamate concentration and protein expression	[104]
13	CUMS	Sprague‒Dawley, male, 2–3 months	SPT, MWM, OFT	Hippocampal LTP	[105]
14	CUMS	Sprague‒Dawley, male, adult (specific age not reported)	SPT, OFT	Hippocampal LTP, protein and mRNA expression	[106]
15	CUMS	Sprague‒Dawley, male, 2–3 months	SPT, MWM	Hippocampal protein expression	[107]
16	CUMS	Sprague‒Dawley, male, 2–3 months	SPT, MWM, OFT, forced swimming test (FST)	Hippocampal protein expression	[108]
17	CUMS	Sprague‒Dawley, male, 2–3 months	SPT, MWM, OFT	Hippocampal protein expression	[109]
18	CUMS	Sprague‒Dawley, male, 60 days	SPT, MWM, FST, home cage locomotion (HCL), exploration and novelty-induced behavior	Hippocampal protein expression	[110]
19	CUMS	Sprague‒Dawley, male, 2–3 months	SPT, MWM, OFT	Hippocampal protein expression	[111]
20	CUMS	Wistar, male, adult (specific age not reported)	SPT, OFT	Hippocampal protein expression	[112]
21	CUMS	Wistar, male, adult (specific age not reported)	SPT, MWM, OFT	Hippocampal protein expression	[113]
22	CUMS	Wistar, male, 7–8 weeks	Sucrose consumption test (SCT), OFT, FST, novelty- induced hypophagia test (NIHP), social interaction test (SIT)	Prefrontal cortex promotor methylation, mRNA and protein expression	[114]
23	CUMS	Sprague‒Dawley, male, 2–3 months	SPT, MWM	Hippocampal LTP	[115]
24	CUMS	Sprague‒Dawley, male, 2–3 months	SPT, OFT, MWM	Hippocampal protein expression	[116]
25	CUMS	Sprague‒Dawley, male, 7–8 weeks	SPT, OFT, FST, MWM	Hippocampal synapse morphometry and protein expression	[117]
26	CUMS	C57BL/6J, male, 4–6 months	SPT, FST, social exploration	Hippocampal mRNA expression	[118]
27	Chronic restraint stress (CRS)	Wistar, male, 8 weeks	FST	Hippocampal neurogenesis and volumetry	[119]
28	CRS	Wistar, male, 7–8 weeks	FST	Hippocampal neurogenesis and volumetry	[120]
29	CRS	Sprague-Dawley, male, (age not reported)	FST	Hippocampal mRNA expression	[121]
31	Chronic water immersion and restraint stress	C57BL/6J, male, 7–8 weeks	Measurement of locomotor activity, FST, NSF	Hippocampal neurogenesis	[122]
31	Chronic social stress (CSS)	C57BL/6J, Male, 10 weeks	Tone-shock fear learning and memory, fear conditioning, treadmill fatigue test, hot plate test	Hippocampal morphometry	[123]
32	Maternal deprivation early in life	Wistar, male and female. Begin of treatment at 60th postnatal day.	Splash test, OFT, FST	Prefrontal cortex and hippocampal protein expression	[124]
33	Surgical model	Sprague‒Dawley, male, 24 weeks	MWM	Hippocampal glutamate concentration and protein expression	[125]
34	Pharmacological model	Wistar, male, adult (specific age not reported)	SPT, FST, Y maze test (YMT), novel object recognition test (NORT)	Hippocampal protein expression	[126]
35	Neuro-endocrine model	Wistar, male, (specific age not reported)	Wet-dog shake behaviors (WDS)	Frontal cortex protein expression	[127]
36	Neuro-endocrine model	Wistar, male, 8–10 weeks	OFT, FST, WDS	Hippocampal protein expression	[128]
37	Neuro-endocrine model	Sprague‒Dawley, male, (specific age not reported)	FST	Frontal cortex and hippocampal mRNA and protein expression	[129]
38	Neuro-endocrine model	ddY, male, 5 weeks	FST	Hippocampal protein expression and morphometry	[130]
39	Neuro-endocrine model	C57BL/6JRj, male, 7–8 weeks	Elevated plus-maze (EPM), NSF, splash test	Peripheral blood mononuclear cell protein expression	[131]
40	Genetic model	Sprague‒Dawley (depressed vs. motivated after selective breeding), male, 60 days	SPT, HCL, FST, EPM (EPM used to test responses to breeding selection, not antidepressant efficacy).	Hippocampal protein expression	[132]
41	Genetic model	Flinders sensitive line (FSL), Flinders resistant line (FRL), Male, adult (specific age not reported)	FST	Hippocampal mRNA expression	[133]
42	Genetic model	FSL, Male, adult (specific age not reported)	FST	Central and peripheral hormone levels	[134]
43	Genetic model	FSL, FRL, male, adult (specific age not reported)	FST	Hippocampal neurogenesis and volumetry	[135]
44	Genetic model	Gunn and Wistar rats, male, 8 weeks	FST, YMT	Prefrontal, limbic and hippocampal morphometry and hippocampal protein expression	[136]
45	Genetic model	Wistar, Wistar Kyoto (WKY), male, 7–8 weeks	FST, OFT, MWM, conditioned emotional response	Cerebral protein expression	[137]
46	Genetic model and CUMS	Wistar, WKY, male, adult (specific age not reported)	SPT, OFT, MWM	Hippocampal protein expression	[138]
47	Genetic and neuro-endocrine model	Microtubule-associated protein 6 knock out mice, C57BL/6 J, male, 2–5 months	FST, NSF	Hippocampal neurogenesis, morphometry and protein expression	[139]
48	Genetic and neuro-endocrine model	hGFAPtk mice (animals with a suppression of neurogenesis in actively dividing GFAP-expressing cells in adulthood) and wild type C57BL/6J, male, 8 weeks	NSF, grooming test, investigation of animal’s coat state	Hippocampal morphometry	[140]

*CUMS* chronic unpredictable mild stress; *CRS* chronic restraint stress; *CSS* chronic social stress; *SPT/SCT* sucrose preference test/sucrose consumption test; *MWM* Morris water maze; *OFT* open field test; *FST* forced swim test; *HCL* home cage locomotion; *NIH/NSF* novelty-induced hypophagia/novelty-suppressed feeding; *YMT* Y maze test; *NORT* novel object recognition test; *WDS* wet dog shake behavior; *EPM* elevated plus maze.

**Fig. 2:**
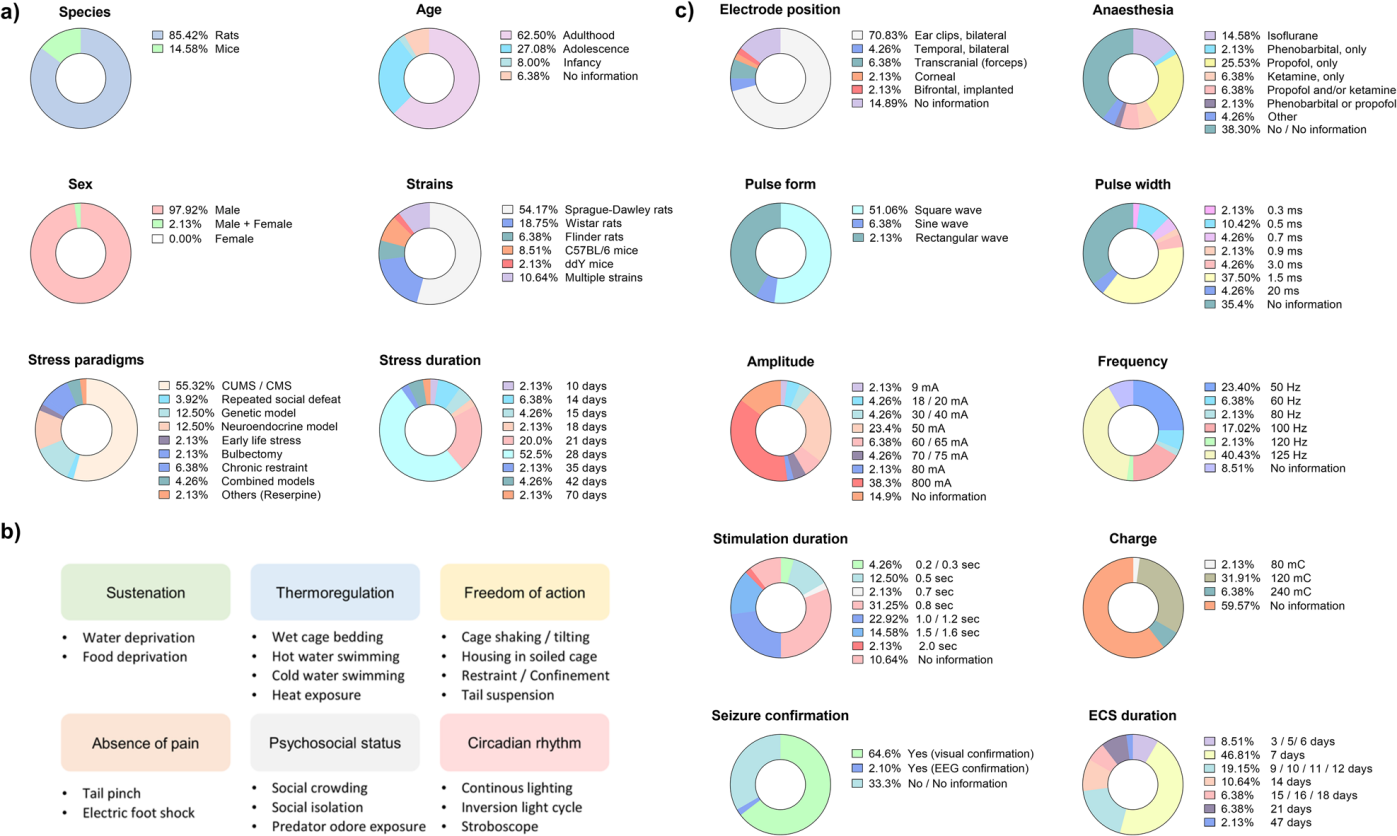
Graphical summaries of the systematic literature review. **a** Distribution of species, strain, age, sex, chronic stress paradigm, and stress duration. **b** Stressor categories and individual stressors employed in the reviewed studies that used CUMS/CMS. **c** ECS details: electrode position, anesthesia, pulse form, width, amplitude, frequency, stimulation duration, charge, seizure confirmation measures, and total ECS duration.

Of the 48 studies, only a minority (n = 7) used mice, while the majority (n = 41) employed rats (Fig. 2a). For both species, almost exclusively male specimens were used: n = 47 males and n = 1 both sexes. No single publication was available that used exclusively female mice or rats. Except for the publication by Ableira et al. 2022, which used an early life stressor in the form of maternal separation in the immediate postnatal period, all the studies used either adolescent (aged ≥ 3 to 60 days) or adult rats (aged ≥ 60 days) or adolescent (aged ≥ 3 to 12 weeks) or adult mice (aged ≥ 12 weeks). In rat studies, the most common strain was Sprague Dawley (n = 26), followed by Wistar (n = 9), the use of multiple strains (n = 5) and Flinder (n = 3) rats. In mouse studies, the majority (n = 4 out of 7) employed only wild-type C57BL/6, while one study used ddY and the remaining two multiple strains. With regard to the stress paradigms employed, n = 26; thus, the majority of the experiments used a chronic stress approach with timewise randomized and modality-wise varying stressors (e.g., water deprivation, social crowding, tail pinch, or isolation) in the form of a CUMS or CMS model. With n = 6 studies each, the two second most common models were genetic and neuroendocrine models. Concerning the latter, most studies have used corticosterone or ACTH injection to mirror HPA axis overactivation. Interestingly, chronic restraint stress (n = 4) and RSDS (n = 1) were rather rare, and only one single study employed bulbectomy to induce depressive-like behaviors. The duration of stress applied ranged between 10 days and 10 weeks, yet three and four weeks were the most common durations (mean: 26 ± 10 days, 95% CI: 22.8 - 29.1 days).

With regard to the CUMS/CMS composition, stressors can be divided into sustenation, thermoregulation, the housing environment, freedom of action, absence of pain, psychosocial status, and circadian rhythm disruption (Fig. 2b). Here, compared to more physical stressors, psychosocial stressors were comparatively underrepresented.

With respect to the ECS, the parameters varied significantly between studies (Fig. 2c). Nonetheless, the most common modalities found were, not considering studies with no information in the respective category: ear clip electrodes (71% of studies, propofol anesthesia (25%), bidirectional square wave pulses (52%) with a 1.5 ms width (38%) and an amplitude of 800 mA (38%) at a frequency of 125 Hz (40%), a stimulation duration of 0.8 seconds (31%), a charge of 120 mC (31%), and 7 days of ECS application (46%). Here it has to be noted, that all studies that have made use 800 mA current intensity in their ECS models originate from China and rely on the Niviqure system. Therefore, this current intensity might be representative of a regional device bias rather than a generally adopted stimulation standard. 67% of the reviewed studies confirmed a tonic‒clonic seizure after ECS. Still, only one study used EEG to confirm seizure induction. Notably, the reporting depth of ECS details varied considerably, which resulted in missing information per category in 9–60% of the studies. Finally, not one study reported the use of muscle relaxation as part of their anesthesia regimen.

To assess behavior, studies have used an array of tests. This included, but was not limited to, the following assessments: SPT/SCT in 58%, the MWM in 54%, the forced swim test (FST) in 46%, the open field test (OFT) in 38%, and novelty-induced hypophagia/novelty suppressed feeding (NIH/NSF) in 10% of studies. Overall, 21% of studies used one, 27% two, and the remaining 52% three or more behavioral tests. On a bulk level, the most impactful effects were the alleviation of anhedonia (odds: 14.5), the restoration of normal locomotion (odds: 6.0), the improvement of despair in the FST (odds: 4.5), and the mitigation of anxiety (odds: 2.0) (Table 2; see Suppl. Table 2 for individual behavioral test results). With regard to memory disturbances, studies have jointly reported a predominantly negative impact of ECS (odds: 0.1). On a biomolecular level, BDNF protein or mRNA levels were the most common readout demonstrating pro-neuroplastic ECS properties in 25% of the investigated studies. Aside from this, no clear effect pattern could be identified due to considerable heterogeneity in the studied targets. Taken together, the reviewed studies demonstrate a predominantly beneficial effect of ECS both on behavioral and molecular changes due to chronic stress.

**Table 2:** Cumulative odds of anhedonia, memory, locomotion, despair, and anxiety according to the behavioral assessments of the reviewed studies.

Behavioral assessment		Total [n] studies	ECS application…			Odds [a/(b + c)]
			(a) improved stress-induced deficits	(b) had no effect on stress-induced deficits	(c) aggravated stress-induced deficits	
**Anhedonia**	SPT/SCT, Splash Test	31	29	2	0	29:2 (**14.5**)
**Memory**	MWM, Y-maze, NORT	29^a^	3	0	26	3:26 (**0.1**)
**Locomotion**	OFT, HCL	21^b^	18	2	1	18:3 (**6.0**)
**Despair**	FST	22^a^	18	4	0	18:4 (**4.5**)
**Anxiety**	NIH/NSF, EPM	6	4	2	0	4:2 (**2.0**)

*SPT/SCT* sucrose preference test/sucrose consumption test, *MWM* Morris water maze, *NORT* novel object recognition test, *OFT* open field test, *HCL* home cage locomotor test, *FST* forced swim test, *NIH/NSF* noveltyinduced hypophagia/novelty-suppressed feeding, *EPM* elevated plus maze.

^a^n = 1 study showed different results for a subpopulation (e.g., male vs. female animals or WKY rats vs. Wistar + CUMS).

^b^n = 1 study was excluded, since it did not use the open field test for any locomotion-related measure.

The 48 publications reviewed must be considered moderately heterogeneous with regard to methodological details such as chronic stress and ECS application, biobehavioral testing and the mechanisms of interest. However, since the findings from both behavioral studies, except for the MWM and Y-maze results, indicate the same direction of beneficial effects on the ECS in mice and rats, the results must be considered homogenous overall in this respect.

**Fig. 3:**
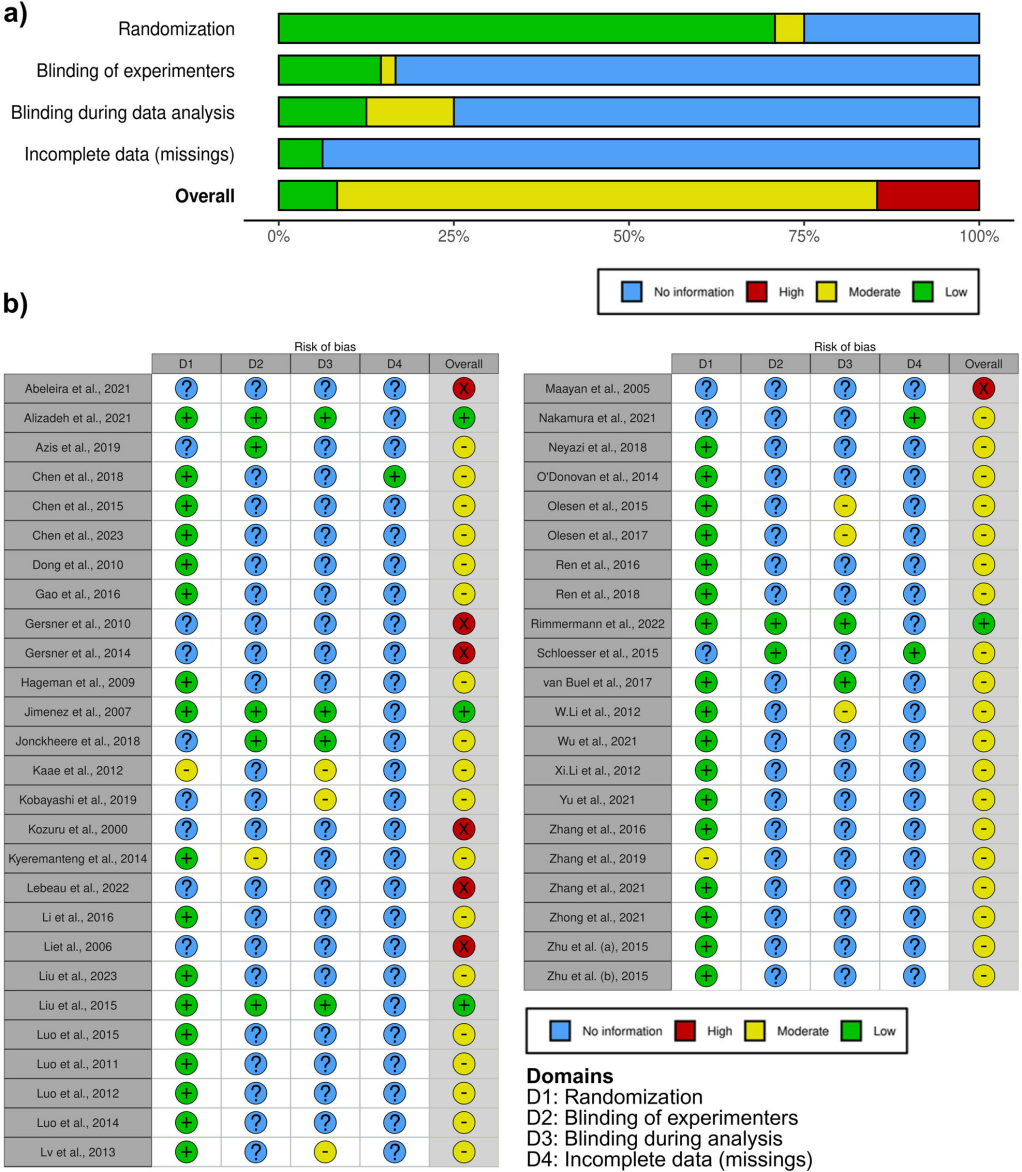
Risk of bias assessment. Risk of bias visualization for all (**a**) and individual (**b**) studies regarding randomization, experimenter and analyst blinding, and missing data.

Concerning the risk of bias, the majority of studies (n = 37) demonstrated an overall moderate risk of bias (Fig. 3a, b). Moreover, 7 studies demonstrated a high and 4 a low bias risk. Interestingly, randomization was a common measure taken by studies, while blinding of experimenters and analysts and reporting of missing data were infrequent.

Finally, considering the number of studies, the extent of odds and directionality of the behavioral findings, and the risk of bias assessment, the authors classified the certainty of the behavioral findings as moderate. Unfortunately, a summarizing evaluation of the certainty of the molecular results was not possible due to the plethora of different targets and systems assessed by various studies.

## Discussion

In our study, we have provided a systematic review of the preclinical body of evidence from murine and rat studies assessing the behavioral and, in parts, biological effects of ECS as a model of ECT in chronic stress-based depression models. The review process revealed that the majority of studies showed that ECS alleviated depressive biobehavioral conditions in rodents. However, several studies have indicated that ECS is closely related to learning and cognitive impairments. In addition to rising or recovering BDNF levels, which have been found both in humans and nonhuman primates [25, 43–45], the biological findings of the reviewed studies were heterogeneous and thus did not allow for any reliable conclusions with regard to the individual behavioral findings on an individual or group level. This can, at least partially, be explained by the methodological variance within the ECS in depression models; the lack of a between-model standard approach to stress and effect success validation in preclinical in vivo research in rodents; and the diversity of the investigated biological mechanism of ECS action assessed. However, the biobehavioral effects of ECS in chronic stressed-based depression models still overlap to a considerable extent with the clinical and biobehavioral effects described in humans with depression undergoing ECT. Overlapping with the behavioral rodent findings in this review, depressive symptoms such as anhedonia, appetite changes, psychomotor symptoms, fatigue, and concentration problems are ameliorated by ECT, while memory, attention, and executive function can be transiently worsened (usually within 3 days of the respective ECT) as a consequence of the triggered seizures [46–48]. However, a large meta-analysis of n = 84 studies did show that cognitive function, following a short-term impairment, improves and exceeds baseline long-term after ECT in depressed patients [48].

Despite the overall translational overlap between the behavioral and symptom findings and directionality between humans and rodents, limitations that should be taken into consideration exist before transferring data about the effects of ECS in rodent depression models to humans. First, despite the undisputable benefits of animal and especially rodent models of diseases [49], they have inherent limitations. On the basis of the evidence reviewed here, small sample sizes, non-existing studies on females despite their epidemiological predominance in depression [12], and incomprehensive behavioral phenotyping limit the generalizability of the findings. This also applies to the chronic stress-based depression models included in our review, of which the most common was CUMS. Here, there was notable variability in the kind and intensity of stressors used to establish these models across studies. Although CUMS/CMS is considered a prototypical example of an animal depression model, its reproducibility can vary as a result of the different overall severities of the stressors applied [40, 50]. Moreover, recent studies have revealed that the effects of CUMS/CMS are primarily linked to inflammation and the immune response at the gene expression level (mRNA) [51]. Additionally, CUMS was linked to proinflammatory cytokine levels (e.g., IL-6), reduced 5-hydroxytryptamine (5-HT) and norepinephrine concentrations, and sex-specific immune changes, such as changes in CD4 and CD8 lymphocyte counts [52]. This insight skews the CUMS/CMS model scope and purviews away from “average” depression and toward depression subtypes involving immune dysregulation [53–55]. In line with these findings, different preclinical rodent models (RSDS, CUMS/CMS, prenatal stress) represent distinct systems biological dimensions of the patho-signature of depression in terms of their molecular validity compared to human postmortem brain samples of depressive patients [56].

In addition, reproducibility challenges arising from heterogeneity, namely methodological variation between studies, are well-recognized issues in animal research [32, 57]. This is primarily due to an overall lack of standardized guidelines and/or coordinated multicenter trials, which results in highly variable experiments [58]. However, preclinical studies are always highly individual and thus variable by nature, as the research objective is commonly more basic than translational. This heterogeneity is often viewed as an issue, yet more recent approaches support the heterogenization of study samples and conditions [59], especially in preclinical research [42]. Nevertheless, in contrast to the multicausal and somewhat arbitrary heterogeneity found in the current literature, a more systematic approach should be taken in the future to increase the robustness of the findings and ultimately increase reproducibility [60]. It could thus be reasoned that the heterogeneity in methodology and findings in the reviewed body of evidence should not necessarily be viewed as a constraint, even in its existing form, but rather as a circumstance more or less corroborating the broad biobehavioral effects and therapeutic power of ECS in different rodent models of the same psychopathology, as demonstrated here for affective spectrum disorder conditions. This argument also maps onto the use of ECT in different environmental stress-associated neuropsychiatric disorders, including but not limited to, depression, mania, or schizophrenia [61]. Moreover, considering the immune-heavy effects of CUMS/CMS, the efficacy of ECS in this review’s body of evidence aligns with the demonstrated efficacy in treating depression with inflammatory features. Although ECT has a strong short-term proinflammatory impact, it appears to support a long-term decrease in inflammatory parameters (e.g., proinflammatory macrophage function) and beneficial changes in mitochondrial energy metabolism accompanied by clinical improvement [62–66]. With regard to methodological standardization, it could be argued that neither rigorous homogenization nor heterogenization are the answer to all problems, yet a combination of both might best cover the complex composition of random and aetiopathological specific effects. Nonetheless, to improve between-model comparability in studies pursuing a similar objective, standardized approaches such as a depression-like syndrome (DLS) could help and serve as a methodological validation tool to increase external validity and thus boost the generalizability of findings. The latter could also significantly amplify the translational value of rodent findings for clinical trials and vice versa [32]. With regard to translatability, the included n = 47 studies all used, for contemporary standards, a rather small test battery for behavioral assessment of the antidepressant effects of ECS, which greatly limits the validity of these findings. This is particularly meaningful since depression is a highly complex clinical condition associated with a great variety of possible symptoms and clusters (i.e., 277 symptom combinations possibly meeting the DMS-IV diagnostic criteria) [67]. To account for this, future trials need to consider more comprehensive and broader approaches to best capture single and cluster behavioral changes in relation to species and strain, sex, stress, and ECS modalities. This approach is imperative, especially since there are multiple highly advanced tools and approaches available (e.g., the deep open field package IntelliCages) [32, 68–71]. In addition, studies should consider increasing sample sizes overall or providing, analogous to clinical trials, power and sample size calculations, including effect size assumptions, to estimate optimal animal numbers per experiment. Moreover, studies must use and prioritize female rodents as well as mixed-sex designs on a regular basis to allow meaningful conclusions to be drawn about sex and sex-stress interaction effects with regard to ECS response.

Another limitation of the reliability but also the translational potential of the included studies is the great variability in ECS application. In humans, ECT is delivered in a controlled clinical setting after the induction of anesthesia and the application of a muscle relaxant [72]. Vital parameters, blood oxygen levels, electrocardiography (ECG) and electroencephalography (EEG) were monitored during ECT. After the induction of anesthesia, a brief electrical stimulus (max. 8 sec) is delivered via cutaneous electrodes to one or both cerebral hemispheres in the form of a series of bidirectional square-wave pulses. This is referred to as a brief (pulse width between 0.5 and 2.0 ms) or an ultrabrief pulse (pulse width below 0.5 ms). The intensity or dose of the stimulus is primarily expressed in terms of the percentage of the applied charge (max. 504 mC, 0–200%) [73–75]. To estimate the appropriate dose, algorithms based on the age and sex of the patient or the method of dose titration, which itself is based on the seizure threshold, can be used. There are three commonly used electrode placements in ECT practice: bilateral (BL) stimulation, also known as bitemporal (BT) stimulation; right unilateral (RUL) stimulation; and bifrontal (BF) stimulation. Notably, the placement of electrodes has been shown to exert a significant effect on the outcome of treatment and the associated side effects [76]. BL stimulation, for example, shows increased clinical efficacy but is also associated with greater side effects [77]. Routine practice involves administering ECT 2–3 times a week [73, 74]. To determine ECT quality, seizure quality indices (SQIs) have been established. SQIs are based on different primary ECT readouts. Commonly, quality is assessed on the basis of five criteria (duration > 25 seconds, postictal suppression > 80%, midictal amplitude >180 μV, intraictal coherence of both hemispheres in the EEG > 90%, tachycardia > 125 bpm) and is sometimes classified as ideal (4–5 criteria), sufficient ( ≥ 3 criteria) or insufficient (≤2 criteria) [75, 78–81]. The number of ECT treatments required to achieve response and/or remission varies between 6 and 15 sessions [72]. According to the reviewed evidence, ECS application is highly inhomogeneous with regard to stimulation parameters, including ECS frequency but also extends to the application details and the utilization of anesthetics. Because anesthetics typically affect the seizure threshold and biochemical processes in the CNS as well as in the periphery, differences in their application can change the effects of ECS. Furthermore, in many but not all studies, rodents were included only if ECS resulted in visible tonic and/or clonic convulsions. This stands in stark contrast to the clinical application of ECT, where anesthesia nearly completely suppresses the tonic‒clonic element in the periphery. Furthermore, visual seizure confirmation, as performed in almost all reviewed studies, is methodologically archaic and should be replaced by more modern techniques, foremost EEG. There are also important differences in electrode placement between rodents and humans. However, in humans, the most common electrode placement is unilateral (e.g., RUL) because it is clinically effective while triggering tolerable cognitive side effects [48, 82]; rodents in the reviewed studies exclusively received bilateral stimulation. This treatment increases antidepressive efficacy but presumably also exacerbates cognitive and memory side effects. This might explain why both memory improvements and disturbances were found in the respective tests (e.g., MWM or Y maze) in the included studies. Additionally, general anesthesia in rodents appears to be associated with cognitive and memory deficits, especially depending on the specific drugs used (e.g., higher after inhalation of anesthetics) and the duration of application [83]. Therefore, the details of ECS and anesthesia, as well as the explicit experimental schedule of stress exposure, ECS, and behavioral testing, may strongly affect the results, especially concerning memory impairment. Another factor that remains to be investigated and clarified is whether not only ECS and anesthesia on their own but also their interaction may modulate the extent and quality of cognitive and memory effects. Because of the differences in the effectiveness and side effects of these two ECT treatments mentioned above and the potentially slightly distinct biological mechanisms of action, the effects of ECS in animal studies might differ somewhat from the effects of ECT in depressed humans. For that reason, which limits the face and construct validity of ECS in depression models [30], future studies should thus aim for more detailed and standardized reporting, for example, via a reporting template; increased uniformity in stimulation with regard to electrode placement; pulse width; voltage; stimulation duration; and confirmation of successful ECS (e.g., visible tonic‒clonic seizure or EMG activity for a defined duration in trials with no muscle relaxation application or EEG derived from implanted or cutaneous electrodes as a confirmation of triggered seizures over a predefined period of time in trials with or without muscle relaxant use). As SQIs have demonstrated clinical feasibility and reliability in predicting ECT success to a certain extent [84–87], an equivalent approach might prove useful for rodent ECS. Here, postictal suppression appears to be one of the most promising single markers since it has been clearly associated with beneficial treatment outcomes [85, 86, 88]. With regard to precise electrode placement, unilateral stimulation paradigms, which, to the best of our knowledge, are currently unavailable, could provide useful information for advancing our understanding of the biological basis of the observed difference in clinical effects between uni- and bilateral stimulation. Furthermore, as electrode placement differs significantly compared to ECT in humans, the construct validity of current approaches in ECS should be called into question. Most studies covered in this review applied auricular electrodes, which may introduce confounders due to issues of anatomical and physiological translatability, e.g., differences in resistance with effects on current delivery and therefore seizure induction. As has already been suggested elsewhere [89], future experiments could, for example, use implanted electrodes at predefined stereotactic coordinates (e.g., analogous to the RUL, placing electrode 1 right anterior above the orbitofrontal cortex and electrode 2 above the apex) or reapply cutaneous electrodes on certain coordinates, for instance, in relation to the bregma, to achieve uniform electrode placement and stimulation conditions, while, moreover, lowering the stimulation energy applied to elicit adequate seizures and minimizing the chances of undesirable electrophysiological, and therefore also molecular, off-target effects, due to topological constraints in electric current delivery. This would drastically boost the translational value and the face and construct validity of the ECS. However, since human trials have demonstrated that unilateral stimulation that is too weak is associated with insufficient ECT efficacy, this has to be factored into the translational process [85]. Concerning anesthesia in general and the use of muscle relaxation agents in particular, the reviewed rodent studies lack some face and construct validity concerning modern ECT setups: not a single of the reviewed studies has employed muscle relaxation in their model. However, the latter is, together with general anaesthesia, a core feature of modern modified ECT, as severe side effects, including bone fractures and muscle strains, were common unwarranted outcomes prior to this technical improvement in the 1940s and 1950 [85]. Interestingly, reviews have shown fractures to be quite common in rat ECS models (12.8% of animals suffer from spinal fractures) [90]. Thus, the reviewed body of evidence actually models a mix of modern modified ECT and an actual tonic‒clonic seizure, including all the decay products and processes associated with postictal inflammation triggered downstream of transient peripheral muscle overactivation, including fracture risk [91, 92]. It remains to be determined which beneficial and side effects repeated severe muscle contractions during ECS cause compared to routine ECT with negligible muscle activation. In conclusion, future studies aiming to translate mechanistic knowledge from rodent models to human ECT and back to clinical applications face the challenge of reproducing the applied ECT parameters, including anesthesia, as closely as possible while simultaneously focusing on ECT-related mechanisms of action from a systems biology perspective. For a summary of suggestions for future rodent ECS studies to improve the translation potential of clinical ECT applications, see Table 3.

**Table 3:** Summary of suggestions for future rodent ECS studies to improve the translation potential of clinical ECT applications.

▪ Include and prioritize female and mixed-sex groups to compensate for the thus far predominantly male geno-/phenotype derived knowledge
▪ Employ comprehensive behavioral and molecular phenotyping (i.e., omics) to fine-granular characterize the link between behavioral and biological ECS treatment effect subtypes – this is particularly important to enable successful bench to bedside translation, and vice versa
▪ Establish a uniform and stable positioning system of ECS electrodes (e.g. via stereotactic coordinates for implantation)
▪ Perform unihemispheric (vs. bilateral) ECS to apprehend the biological mechanisms that underlie effect/side-effect profile differences
▪ Use and report anesthesia (in particular: muscle relaxation) to upgrade face and construct validity concerning a modern ECT setup
▪ Improve bias reduction measures (e.g., reduction of missings) as well as reporting on potential risks of bias in the publication itself
▪ Use larger, that is adequate, sample sizes per group/sex/experiment, ideally based on prior effect size and power calculations
▪ Develop and use a standardized reporting system (e.g. template) of ECS stimulation parameters to increase between-study comparability
▪ Report stimulation success for each ECS session (ideally: incorporate intraictal EEG – minimal criterion, only if EEG is not available: visual confirmation of tonic-clonic seizure), ideally in the form of a rodent SQI
▪ For translational studies: adhere to between-model standardization approaches for stress application (e.g., similar CUMS/CMS stressor sequence) and biobehavioral phenotype/subtype validation per animal and group (e.g., depression-like syndrome framework)
▪ For select research objectives: consider systematic heterogenization and larger, multicentered trials to improve the robustness of findings

*ECS* electroconvulsive shock, *ECT* electroconvulsive therapy, *EEG* electroencephalogram, *SQI* seizure quality index, *CUMS/CMS* chronic unpredictable mild stress/chronic mild stress.

Finally, since preclinical studies are highly experimental by nature, a moderate risk of bias and confidence in the reviewed literature should be considered satisfactory with regard to the demonstrated methodological heterogeneity, different research objectives and aetiopathological targets of interest. However, it is admittedly out of the question that a lower risk of bias as well as greater confidence would strengthen preclinical ECT research. In addition, improved reporting and the deposition of animal data in online repositories would enforce the compilation of both systematic effect size calculations and meta-analyses.

To the best of our knowledge, this is the first ever systematic review of preclinical studies assessing the biobehavioral effects of ECS in rodent models of depression in the context of chronic stress exposure. In conclusion, within the conceptual limits of rodent-to-human translation, the compiled evidence underlines the therapeutic power and broad beneficial effects of ECS as a preclinical equivalent of ECT in rodent depression research and overlaps in directionality and quality of beneficial effects with the symptom improvements observed in depressed patients. Nonetheless, methodological improvements, including the translational impact of this preclinical technique, are key to potentiate internal and external validity.

## Supplementary information

Supplementary Table 1: Detailed overview of stress application, ECS parameters and biobehavioral outcomes including methods and statistics for all reviewed studies.

Supplementary Table 2: Distribution of ECS effects per behavioral test and study, subdivided into beneficial, no, or adverse impact on animal phenotypes.
