# Supplementary material for: Contemporary in vivo rodent electroconvulsive therapy (ECT) models in translational depression research: a systematic review
Source: Transl Psychiatry. 2025 Nov 29;15:515. doi: 10.1038/s41398-025-03749-x (PMC12669789; doi:10.1038/s41398-025-03749-x)
Supplement: Supplementary file 1 — Supplementary Table 1: Detailed overview of stress application, ECS parameters and biobehavioral outcomes including methods and statistics for all reviewed studies. [file 41398_2025_3749_MOESM1_ESM.pdf]

Supplementary Table 1: Detailed overview of stress application, ECS parameters and biobehavioral outcomes including methods and statistics for all reviewed studies.

Abbreviations: (2R,6R)-HNK = (2R,6R)-hydroxynorketamine; 5-HT-2A = serotonin 2A-receptor; ACTH = adrenocorticotropic hormone; ANOVA = analysis of variance; ASC = apoptosis-associated speck-like protein containing a CARD; Aβ = amyloid-β peptide; BDNF = brain-derived neurotrophic factor; BP = brief pulse; BrdU = 5-bromo-2'-deoxyuridine; CaMKIIα = Ca^{2+}/calmodulin-dependent kinase II α isoform; CAPON = carboxy-terminal PDZ ligand of nNOS; CB1R = cannabinoid receptor type 1; CER = conditioned emotional response; CMS = chronic mild stress; CORT = corticosterone; CREB = cAMP-response element-binding protein; CRF = corticotropin-releasing factor; CRS = chronic restraint stress; CSS = chronic social stress; CUMS = chronic unpredictable mild stress; CUS = chronic unpredictable stress; DCX = doublecortin; DEX = dexmedetomidine; DG = dentate gyrus; DHEA = dehydroepiandrosterone; DMSO = dimethyl sulfoxide; DOI = (+/-)-1-(2,5-dimethoxy-4-iodophenyl)-2-aminopropane; Drd1 = dopamine D1 receptor; DRL = depressed rat line; ECS = electroconvulsive shock; EdU = 5-ethynyl-20-deoxyuridine; EL = escape latency; EPM = elevated plus-maze; fEPSP = field excitatory postsynaptic potential; Flx-NR = fluoxetine-non-responding; Flx-R = fluoxetine-responding; FRL = Flinders resistant line; FSL = Flinders sensitive line; FST = forced swimming test; GABA = γ-Aminobutyric acid; $GABA_AR$ = GABA receptor subunit A; GAD65 = glutamic acid decarboxylase 65; GCL = granule cell layer; GFAP = glial fibrillary acidic protein; GluN = phospho-NMDA receptor; $GluR_1$ = glutamate receptor subunit 1; HCL = home cage locomotion; i.p. = intraperitoneal; Iba-1 = allograft inflammatory factor 1; IL = interleukin; LC = microtubule-associated protein light chain; LSD = least significant difference; LTP = long-term potentiation; mBDNF = mature BDNF; MECS = modified ECS; MWM = Morris water maze; NAC = nucleus accumbens; NIHP = novelty-induced hypophagia test; NLRP3 = NOD-like receptor family pyrin domain-containing protein 3; NMDA = N-methyl-D-aspartate receptor; nNOS = neuronal nitric oxide synthase; NORT = novel object recognition test; NPY = neuropeptide Y; NR2B = NMDAR subtype 2B; NSF = novelty-supressed feeding test; OF = open-field test; PAI-1 = plasminogen activator inhibitor-1; PK2 = prokineticin 2; PLC = prelimbic cortex; PSD-95 = postsynaptic densities-95; PST = Porsolt swim test; s.c. = subcutaneous; SCES = subconvulsive electrical stimulation; SCT = sucrose consumption test; SET = space exploration time; SIT = social interaction test; SNK = Student–Newman–Keuls; SPP = sucrose preference percentage; SPT = sucrose preference test; SYP = synaptophysin; TBRAS = thiobarbituric acid reactive species; TNF = tumor necrosis factor; tPA = tissue plasminogen activator; UBP = ultrabrief pulse; VGCV = valganciclovir; vPLC = ventral prelimbic cortex; VTA = ventral tegmental area; WDS = wet-dog shake; WKY = Wistar Kyoto rats; YMT = Y maze test

	Stress paradigm<br>Country of origin	Stress application, duration	Strain, sex, age	ECS application and timing	Behavioural assessment	Main behavioural and molecular outcomes	Ref
**1**	Chronic unpredictable mild stress (CUMS)<br><br>China	Stressors were applied randomly. Stressors included: water deprivation for 24 h; food deprivation for 24 h; tailing pinching for 1 min; shaking for 20 min; social crowding for 24 h; continuous lighting for 24 h; cold water swimming at 4°C for 5 min; hot water swimming at 45°C for 5 min; damp sawdust for 24 h; cage tilting (45°) for 24 h.<br><br>Daily for 28 days.	Sprague-Dawley<br><br>Male, 2–3 months	Administration via bilateral ear clip electrodes with a Nivique ECS system. Parameter details: bidirectional square wave pulses, 0.8 A amplitude, 1.5 ms width, 125 Hz frequency, 0.8 s duration and 120 mC charge. Sham ECS was conducted procedurally similar, yet without current administration. Anaesthesia: intraperitoneal (i.p.) injection of propofol (80 mg/kg) or saline (8 ml/kg).<br><br>Daily for 7 days.	Sucrose preference test (SPT),<br>Morris water maze (MWM)	The sucrose preference percentage (SPP) of ECS-treated groups (saline and propofol) was higher compared to sham+saline (n=6/group, all $p<0.05$, repeated-measures ANOVA). After both ECS+saline and ECS+propofol, rats exhibited prolonged escape latency (EL) in the MWM (n=6/group, all $p<0.05$, repeated-measures ANOVA). The EL after ECS+saline was longer than ECS+propofol (n=6/group, all $p<0.05$, repeated-measures ANOVA). After ECS, the space exploration time (SET) in the MWM of ECS+saline group was the shortest (n=6/group, $p<0.05$, repeated-measures ANOVA). Comparably, the SET of the ECS+propofol rats was longer (n=6/group, $p=0.003$, post hoc tests). ECS induced long-term potentiation (LTP) impairment compared to sham ECS+saline (n=6/group, $p<0.05$, post hoc tests). The impairment of LTP induced by ECS was attenuated in the ECS+propofol group (n=6/group, $p<0.05$, one-way ANOVA). Propofol decreased the ECS-induced expression of hippocampal p-T305-CaMKII (n=6/group, $p<0.05$, post hoc tests) and increased the down-regulated expression in hippocampal postsynaptic densities (PSDs) (n=6/group, $p=0.013$, one-way ANOVA).	[1]
**2**	CUMS<br><br>China	Stressors were applied randomly. Stressors included: swimming for 5 min in 4 °C cold water; swimming for 5 min in 45 °C hot water; tail pinching for 1 min; food deprivation for 24 h; water deprivation for 24 h; shaking for 20 min; continuous lighting for 24 h; damp sawdust for 24 h; cage tilting (45°) for 24 h.<br><br>Daily for 28 days.	Sprague-Dawley<br><br>Male, 2–3 months	Administration via bilateral ear clip electrodes with a Nivique ECS system. Parameter details: bidirectional square wave pulses, 0.8 A amplitude, 1.5 ms width, 125 Hz frequency, 0.8 s duration and 120 mC charge. Sham ECS was conducted procedurally similar, yet without current administration. Oxygen was provided and oxygen saturation was monitored during ECS.	SPT,<br>MWM	In the SPT, SPP after ECS was higher in the DE5 (CUMS+5 ECS) and DE7 (CUMS+7 ECS) group compared to the CUMS+sham ECS group (n=9/group, $p<0.01$ respectively, one-way ANOVA followed by the Student–Newman–Keuls (SNK)-q test). SPP after ECS was higher in the DE5 and DE7 group compared to DE1 or DE3 group (n=9/group, $p<0.05$ each, one-way ANOVA followed by the SNK-q test). There was no significant difference in the SPP after ECS between the CUMS+sham ECS group and DE1 (CUMS+1 ECS) or DE3 (CUMS+3 ECS) group (n=9/group, $p>0.05$ respectively, one-way ANOVA followed by the SNK-q test).<br>In the MWM, the EL after ECS was longer in the DE5 and DE7 groups compared to the D group (n=9/group, $p<0.05$ respectively, repeated-measures ANOVA). EL was higher in the DE5 and DE7 groups compared to DE1 or DE3 groups (n=9/group, $p<0.05$ each, repeated-measures ANOVA). There was no significant difference in the EL between the CUMS+sham ECS group and DE1 or DE3 groups (n=9/group, $p>0.05$ respectively, repeated-measures ANOVA).	[2]

				All rats treated with ECS showed tonic-clonic seizures with a duration of at least 10 sec.<br>Anaesthesia: i.p. injection of propofol (90 mg/kg).<br><br>Daily for 1 (group DE1), 3 (group DE3), 5 (group DE5) or 7 (group DE7) days.		In the MWM, the SET after ECS was shorter in the DE5 and DE7 group compared to CUMS+sham ECS group (n=9/group, p<0.05 respectively, one-way ANOVA followed by the SNK-q test). SET was shorter in the DE5 and DE7 group compared to DE1 or DE3 group (n=9/group, p<0.05 each, one-way ANOVA followed by the SNK-q test). There was no significant difference in the SET between the CUMS+sham ECS group and DE1 or DE3 group (n=9/group, p>0.05 respectively, one-way ANOVA followed by the SNK-q test).<br>The baseline field excitatory postsynaptic potential (fEPSP) in the hippocampal Schaffer collaterals-CA1 pathway was higher in the DE3, DE5 and DE7 groups compared to the CUMS+sham ECS group (n=6/group, p<0.01 each, one-way ANOVA followed by the SNK-q test). There was no significant difference in the fEPSPs between the CUMS+sham ECS group and DE1 group (n=6/group, p>0.05, one-way ANOVA followed by the SNK-q test). fEPSP was higher in the DE5 and DE7 group compared to DE1 or DE3 groups (n=6/group, p<0.05 respectively, one-way ANOVA followed by the SNK-q test).<br>LTP in the hippocampal Schaffer collaterals-CA1 pathway was lower in the DE3, DE5 and DE7 groups compared to CUMS+sham ECS group (n=6/group, p<0.01 each, one-way ANOVA followed by the SNK-q test). There was no significant difference in the LTP between the CUMS+sham ECS group and DE1 group (n=6/group, p>0.05, one-way ANOVA followed by the SNK-q test). LTP was lower in the DE3 and DE5 group compared to DE1 or DE3 groups (n=6/group, p<0.05 each, one-way ANOVA followed by the SNK-q test).	
**3**	CUMS<br><br>China	Stressors were applied randomly.<br>Stressors included: continuous lighting for 24 h; tail pinching for 1 min; cage tilting (45°); cold water swimming at 4°C for 5 min; damp sawdust bedding for 24 h; water deprivation for 24 h; food deprivation for 24 h; shaking (1 shake/s) for 20 min; social crowding for 24 h.<br><br>Daily for 28 days.	Sprague-Dawley<br><br>Male, 2–3 months	Administration via bilateral ear clip electrodes with a Niviqure ECS system.<br>Parameter details: bidirectional square wave pulses, 0.8 A amplitude, 1.5 ms width, 125 Hz frequency, 0.8 s duration and 120 mC charge.<br>A tonic-clonic seizure with a duration of at least 10 s was used as an ECS success marker.<br>Anaesthesia: i.p. injection of propofol (80 mg/kg) or saline (8 ml/kg).	SPT, MWM	Rats receiving propofol+ECS showed shorter seizure duration compared to rats receiving saline+ECS (n=18/group, p<0.05, ANOVA).<br>In SPT, SPP was higher in the rats receiving propofol+ECS and saline+ECS compared to rats receiving saline without ECS (n=18/group, p<0.05 each, ANOVA). There was no significant difference in the SPP between rats receiving propofol+ECS and saline+ECS (n=18/group, p=0.736, ANOVA).<br>In the MWM, the EL of rats receiving propofol+ECS and saline+ECS was longer compared to the rats receiving saline without ECS (n=18/group, all p<0.05, ANOVA). The EL of the rats receiving propofol+ECS was shorter compared to the rats receiving saline+ECS (n=18/group, p<0.05, ANOVA). Rats receiving saline+ECS showed shorter SET compared with rats receiving propofol+ECS (n=18/group, p<0.05, ANOVA).<br>Rats receiving saline+ECS showed an impairment of hippocampal LTP compared to rats receiving saline without ECS (n=5-6/group, p<0.05, ANOVA). Rats receiving propofol+ECS exhibited an alleviated LTP impairment compared to rats receiving saline+ECS (n=5-6/group, p<0.05, ANOVA).	[3]

				Daily for 7 days.		The hippocampal expression of mBDNF, proBDNF and the proBDNF/mBDNF ratio in rats receiving saline+ECS was higher compared to rats receiving saline without ECS (n=6/group, p<0.05 each, ANOVA). The hippocampal expression of mBDNF was higher, but proBDNF and the proBDNF/mBDNF ratio were lower in rats receiving propofol+ECS compared to rats receiving saline+ECS (n=6/group, p<0.05 each, ANOVA).<br>The expression of hippocampal tPA was higher in rats receiving saline+ECS compared to rats receiving saline without ECS (n=6/group, p<0.05, ANOVA). Rats receiving propofol+ECS showed a higher level of hippocampal tPA expression compared to rats receiving saline+ECS (n=6/group, p<0.05, ANOVA). There was no significant difference in the hippocampal PAI-1 expression between rats receiving saline+ECS and propofol+ECS compared to rats receiving saline without ECS (n=6/group, p=0.351 and p=0.194 respectively, ANOVA).	
**4**	CUMS<br><br>China	Stressors were applied randomly. Stressors included: cold water swimming at 4°C for 5 min; hot water swimming at 4°C for 5 min; continuous lighting for 24 h; damp sawdust for 24 h; water deprivation for 24 h; food deprivation for 24 h; cage tilting (45°) for 24 h; stroboscopic light for 4 h; tail pinching for 1 min; shaking for 20 min.<br><br>Daily for 28 days.	Sprague-Dawley<br><br>Male, 2-3 months	Electrode placement and ECS device were not reported. Parameters details: bidirectional square wave pulses, 0.8 A amplitude, 1.5 ms width, 125 Hz frequency. 0.4, 0.8, 1.2 and 1.6 s duration for 60, 120, 180, and 240 mC charge, respectively. Duration and charge varied among the experimental groups (M60, M120, M180, M240).<br>Sham ECS (group M0) was conducted procedurally similar, yet without current administration.<br>Anaesthesia: i.p. injection of propofol (80 mg/kg).<br><br>Daily for 7 days.	SPT,<br>MWM,<br>Open field test (OF)	In the SPT, SPP was higher for the groups M120, M180, M240 compared with sham ECS and M60 (n=6/group, p<0.001 each, ANOVA). There was no significant difference between sham ECS and M60 and M120, M180 and M240 (n=6/group, p>0.05 each, ANOVA).<br>In the MWM, EL was longer with the increase of charge (n=6/group, p<0.05, ANOVA), but there was no significant difference between sham ECS and M60 or M180 and M240 groups (n=6/group, p>0.05, ANOVA). SET shortened with the increase of charge (n=6/group, p<0.05, ANOVA), but there was no significant difference between sham ECS and M60 or M120 and M180 groups (n=6/group, p>0.05 respectively, ANOVA).<br>In the OF, incidence of rearing was higher in the M120, M180 and M240 groups compared to sham ECS and in the groups M180 and M240 compared to M60 (n=6/group, p<0.05 each, ANOVA). The amount of grid crossing was higher for the groups M120, M180, M240 compared with sham ECS and M60 (n=6/group, p<0.05 each, ANOVA).<br>There was no significant difference in the expression levels of CaMKIIa in hippocampus between sham ECS, M60, M120, M180 and M240 (n=6/group, p>0.05 respectively, ANOVA).<br>PSD-95 expression levels in the hippocampus were higher in M60, M120, and M180 compared to sham ECS and M240 (n=6/group, p<0.05 each, ANOVA).<br>The hippocampal expression level of GluN1 was lower in group M240 compared with sham ECS, M60, M120 and M180 (n=6/group, p<0.05 respectively, ANOVA).<br>The hippocampal expression level of GluN2A and GluN2B in the sham ECS group was significantly higher than that in the other groups (n=6/group, p<0.05 respectively, ANOVA).	[4]

**5**	CUMS<br><br>China	Stressors were applied randomly. Stressors included: food deprivation for 24 h; water deprivation for 24 h; shaking for 20 min (1 shaking/s); tail pinching for 1 min; damp sawdust for 24 h; continuous lighting for 24 h; social crowding; cold water swimming at 4°C for 5 min; cage tilting (45°) for 24 h.<br><br>Daily for 28 days.	Sprague-Dawley<br><br>Male, 2-3 months	Administration via bilateral ear clip electrodes with a Niviqure ECS system. Parameter details: bidirectional square wave pulses, 0.8 A amplitude, 1.5 ms width, 125 Hz frequency, 0.8 s duration and 120 mC charge. Sham ECS was conducted procedurally similar, yet without current administration. A tonic-clonic seizure was used as an ECS success marker. Anaesthesia: i.p. injection of propofol (80 mg/kg) or saline (8 ml/kg).<br><br>Daily for 7 days.	SPT, MWM	In the SPT, SPP after saline+ECS and propofol+ECS were higher compared to SPP before ECS and compared to saline+sham ECS (n=12/group, all p<0.05, ANOVA). SPP for propofol+ECS rats was lower than for saline+ECS rats and lower than the control (healthy rats without treatment) group (n=12/group, p<0.05 each, ANOVA). There was no significant difference between the control group and rats after saline+ECS treatment (n=12/group, p=0.935, ANOVA).<br>In the MWM, saline+ECS and propofol+ECS groups showed spatial learning and memory impairment with longer EL and shorter SET compared to the saline+sham ECS group (n=12/group, all p<0.05, ANOVA). Propofol+ECS group showed shorter EL and longer SET compared to saline+ECS (n=12/group, p<0.05 each, ANOVA).<br>Saline+ECS and propofol+ECS groups exhibited higher hippocampal expression level of IL-1β and TNF-α compared to the control group (n=6/group, all p<0.05, ANOVA). Hippocampal IL-1β expression levels were higher in the saline+ECS and propofol+ECS group compared to saline+sham ECS group (n=6/group, p<0.05 respectively, ANOVA). Hippocampal TNF-α expression levels were higher in the saline+ECS group compared to saline+sham ECS group (n=6/group, p<0.05, ANOVA), but there was no significant difference between the propofol+ECS and saline+sham ECS group (n=6/group, p=0.247, ANOVA). The propofol+ECS group showed a lower hippocampal expression of IL-1β and TNF-α compared to the saline+ECS group (n=6/group, p<0.05 respectively, ANOVA).<br>The hippocampal expression levels of glutamate aspartate transporter displayed no significant difference between groups (n=6/group, p=0.087, ANOVA). The propofol+ECS group showed higher hippocampal expression levels of glutamate transporter 1 compared to saline+ECS group (n=6/group, p<0.05, ANOVA). The hippocampal glutamate concentration of the saline+ECS group was higher compared to the saline+sham ECS group (n=6/group, p<0.05, ANOVA). The hippocampal glutamate concentration of the propofol+ECS group was lower compared to the saline+ECS group (n=6/group, p<0.05, ANOVA). There was no significant difference between the propofol+ECS and saline+sham ECS group (n=6/group, p>0.05, ANOVA).	[5]
**6**	CUMS<br><br>China	Stressors were applied randomly. Stressors included: water deprivation for 24 h; food deprivation for 24 h; tail pinching for 1 min; damp sawdust for 24 h; shaking (1 shaking/s) for 20 min; continuous	Sprague-Dawley<br><br>Male, 2-3 months	Administration via bilateral ear clip electrodes with a Niviqure ECS system. Parameter details: 0.8 A amplitude, 1.5 ms width, 125 Hz frequency, 0.8 s duration and 120 mC charge.	SPT, MWM	In the SPT, SPP was higher in the ECS and ketamine+ECS groups compared to sham ECS (n=6/group, p<0.05 respectively, ANOVA followed by Bonferroni correction). There was no significant difference between ECS and ketamine+ECS (n=6/group, p=0.124, ANOVA).<br>In the MWM, EL of the rats in the ECS and ketamine+ECS group was longer compared to the rats in the sham ECS group (n=12/group, p<0.05 respectively, ANOVA). SET of the rats in the ECS and ketamine+ECS group was shorter compared to the rats in the sham ECS group (n=12/group, p<0.05 respectively, ANOVA). The rats in the ketamine+ECS group exhibited a shorter	[6]

		lighting for 24 h; social crowding; cold water swimming at 4°C for 5 min; heat stress in an oven at 45°C for 5 min; cage tilting (45°) for 24 h.<br><br>Daily for 28 days.		Sham ECS was conducted procedurally similar, yet without current administration. Anaesthesia: i.p. injection of ketamine (100 mg/kg).<br><br>Daily for 7 days.		EL and longer SET compared to the ECS group (n=12/group, $p<0.05$ respectively, ANOVA).<br>The rats in the ECS group exhibited higher expression levels of Iba-1 in the hippocampal CA1 and DG areas compared to sham ECS (n=6/group, $p<0.05$ respectively, ANOVA). The rats in the ketamine+ECS group showed lower expression levels of Iba-1 in these hippocampal areas compared to the ECS group (n=6/group, $p<0.05$ respectively, ANOVA). There was no significant difference in the expression levels of Iba-1 in the same hippocampal areas between the ketamine+ECS and sham ECS groups (n=6/group, $p>0.05$ respectively, ANOVA).<br>The rats in the ECS group exhibited higher mRNA levels of IL-1β and TNF-α in the hippocampus compared to sham ECS (n=6/group, $p<0.05$ respectively, ANOVA). The rats in the ketamine+ECS group showed lower mRNA levels of IL-1β and TNF-α in the hippocampus compared to the ECS group (n=6/group, $p<0.05$ respectively, ANOVA).<br>The rats in the ECS group exhibited higher hippocampal levels of Aβ1-40 and Aβ1-42 compared to sham ECS (n=6/group, $p<0.05$ respectively, ANOVA). The rats in the ketamine+ECS group showed lower hippocampal levels of Aβ1-40 and Aβ1-42 compared to the ECS group (n=6/group, $p<0.05$ respectively, ANOVA).	
**7**	CUMS<br><br>China	Stressors were applied randomly. Stressors included: food deprivation for 24 h; water deprivation for 24 h; tail pinching for 1 min; continuous exposure to light for 24 h; cold water swimming at 4°C for 5 min; hot water swimming at 45°C for 5 min; shaking for 20 min; damp sawdust for 24 h; cage tilting to 45° for 24 h.<br><br>Daily for 28 days.	Sprague-Dawley<br><br>Male, 2–3 months	Administration via bilateral ear clip electrodes with a Niviqure ECS system. Parameter details: bidirectional square wave pulses, 0.8 A amplitude, 1.5 ms width, 125 Hz frequency, 0.8 s duration and 120 mC charge. Sham ECS was conducted procedurally similar, yet without current administration. Oxygen was provided through mask and oxygen saturation was monitored during ECS. Anaesthesia: i.p. injection of saline (8 ml/kg) or propofol (80 mg/kg) or/and ketamine (10 mg/kg).<br><br>Daily for 7 days.	SPT, MWM	ECS and ECS under propofol and/or ketamine increased the SPP in the SPT (n=10/group, all $p<0.05$, repeated-measures ANOVA). In the MWM, EL was longer and SET was shorter in the ECS+saline group than in ECS+ketamine and ECS+propofol (n=10/group, all $p<0.05$, repeated-measures ANOVA). Rats after ECS+saline treatment showed lower LTP compared to rats after ECS+propofol or ECS+ketamine treatment (n=6/group, all $p<0.05$, paired t test).	[7]

**8**	CUMS<br><br>China	Stressors were applied randomly. Stressors included: cold water swimming at 4°C for 5 min; hot water swimming at 45°C for 5 min; food deprivation for 24 h; water deprivation for 24 h; shaking for 15 min (1 shake/s); continuous lighting for 24 h; tail pinching for 1 min; cage titling to 45° for 24 h; housing in an isolated cage for 24 h.<br><br>Daily for 28 days.	Sprague-Dawley<br><br>Male, 2–3 months	Administration via bilateral ear clip electrodes with a Niviqure ECS system. Parameter details: bidirectional square wave pulses, 0.8 A amplitude, 1.5 ms width, 125 Hz frequency, 0.8 s duration and 120 mC charge. For sham ECS, bitemporal electrode placement without current administration was used. Anaesthesia: i.p. injection of ketamine (10 mg/kg) or propofol (80 mg/kg) or ketamine (10 mg/kg) and propofol (80 mg/kg) or saline (8 ml/kg).<br><br>Daily for 7 days.	SPT, MWM, OF	In the SPT, ECS, ECS+ketamine and ECS+propofol increased the SPP compared to sham treatment (n=10/group, $p<0.05$ each, repeated-measures ANOVA followed by SNK-q test). The SPP after ECS+ketamine+propofol was higher compared to the above-mentioned four groups (n=10/group, all $p<0.05$, repeated-measures ANOVA followed by SNK-q test). In the OF, the total distance travelled and the incidence of rearing of rats after ECS, ECS+ketamine and ECS+propofol were higher than of rats after sham treatment (n=10/group, total distance travelled: $p<0.05$ each, number of rearing: $p<0.05$ each, repeated-measures ANOVA followed by SNK-q test). The total distance travelled and the incidence of rearing after ECS+ketamine+propofol were higher compared to the four above-mentioned groups (n=10/group, total distance travelled: $p<0.05$ each, number of rearing: $p<0.05$ each, repeated-measures ANOVA followed by SNK-q test). In the MWM, ECS treated rats displayed spatial learning and memory impairment with increased EL and reduced SET compared to sham treatment (n=10/group, $p<0.05$, respectively, repeated-measures ANOVA followed by SNK-q test). Compared to the ECS group, EL was lower and SET was higher after ECS+ketamine, ECS+propofol and ECS+ketamine+propofol (n=10/group, $p<0.05$ each, repeated-measures ANOVA followed by SNK-q test). ECS, ECS+propofol and ECS+ketamine+propofol increased the protein expression of the hippocampal p-GluR1 and p-$GABA_A$R compared to sham treatment (n=10/group, $p<0.05$ each, one-way ANOVA followed by SNK-q test).	[8]
**9**	CUMS<br><br>China	Stressors were applied randomly. Stressors included: food deprivation for 24 h; water deprivation for 24 h; cold water swimming at 4°C for 5 min; hot water swimming at 45°C for 5 min; shaking for 15 min (1 shake/s); tail pinching for 1 min; continuous lighting for 24 h; housing in a soiled cage for 24 h; cage titling to 45° for 24 h.<br><br>Daily for 28 days.	Sprague-Dawley<br><br>Male, 2–3 months	Administration with a Niviqure ECS system. Electrode placement not reported. Parameter details: bidirectional square wave pulses, 0.8 A amplitude, 1.5 ms width, 125 Hz frequency, 0.8 s duration and 120 mC charge. For sham ECS, bitemporal electrode placement without current administration was used. Anaesthesia: i.p. injection of propofol (80 mg/kg) or saline (8 ml/kg).<br><br>Daily for 7 days.	SPT, MWM, OF	In the SPT, ECS and ECS+propofol increased the SPP compared to sham treatment (n=10/group, $p<0.05$ respectively, repeated-measures ANOVA followed by Bonferroni correction). There was no significant difference between the ECS and ECS+propofol groups (n=10/group, $p>0.05$, repeated-measures ANOVA followed by Bonferroni correction). In the OF, the total distance and incidence of rearing after ECS and after ECS+propofol increased compared to sham treatment (n=10/group, $p<0.05$ respectively, repeated-measures ANOVA followed by Bonferroni correction). There was no significant difference between the ECS and ECS+propofol groups (n=10/group, $p>0.05$ respectively, repeated-measures ANOVA followed by Bonferroni correction). In the MWM, ECS displayed spatial learning and memory impairment with increased EL and reduced SET compared to sham treatment (n=10/group, $p<0.05$ respectively, repeated-measures ANOVA followed by Bonferroni correction). Propofol alleviated these impairments with decreased EL and increased SET after ECS+propofol compared to ECS (n=10/group, $p<0.05$ respectively, repeated-measures ANOVA followed by Bonferroni correction). After ECS, an increase in the mean density of nNOS, a decrease in the mean density of CAPON, and an increase in the ratio of nNOS/CAPON in hippocampal areas was observed (n=10/group, $p<0.05$ for CA1, CA3 and DG respectively, one-way	[9]

						ANOVA followed by Bonferroni correction). Compared to ECS, the ECS+propofol group exhibited lower expression of nNOS and higher expression of CAPON in the same hippocampal areas (n=10/group, p<0.05 for CA1, CA3 and DG respectively, one-way ANOVA followed by Bonferroni correction) and a decreased nNOS/CAPON ratio (n=10/group, p<0.05 for CA1, CA3 and DG respectively, one-way ANOVA followed by Bonferroni correction).	
**10**	CUMS<br><br>China	Stressors were applied randomly. Stressors included: cold water swimming at 8–10°C for 5 min; tail pinching for 1 min; food deprivation for 24 h; water deprivation for 24 h; social crowding with cage tilted (30°) for 24 h; shaking for 20 min (1 shake/s); continuous lighting for 24 h; housing in an isolated cage for 24 h; heat stress in oven at 42°C for 5 min; undesirable confinement for 2 h.<br><br>Daily for 21 days.	Sprague-Dawley<br><br>Male, (specific age not reported)	Administration via electrodes (bitemporal placement) with a DX-II pulse generator. Parameter details: 0,05 A current, 50 Hz frequency, 1 s duration. Sham ECS was conducted procedurally similar, yet without current administration. Oxygen saturation was monitored during ECS. Anaesthesia: i.p. injection of propofol (100 mg/kg), pentobarbital sodium (2%, 45 mg/kg), or saline (10 ml/kg). Duration and intensity of the seizures induced by ECS was assessed by EEG for a part of the experiments.<br><br>Every other day for 12 days.	SPT, MWM, OF	ECS and ECS under propofol and pentobarbital sodium increased SPP in the SPT and increased the ambulation and rearing scores in the OF (n=12/group, p<0.05 each, repeated-measures ANOVA). ECS increased the EL and decreased the percentage of dwell time (n=12/group, p<0.05 each, repeated-measures ANOVA). EL was shorter and the percentage of dwell time was longer under propofol or pentobarbital sodium (n=12/group, p<0.05 each, repeated-measures ANOVA). There was no significant difference between the antidepressant effect of ECS under saline, propofol and pentobarbital sodium as measured in the SPT and OF (n=12/group, p>0.05, repeated-measures ANOVA). ECS+saline downregulated the ratio of Glu/GABA (n=6/group, p<0.05, ANOVA) and upregulated GAD65 expression in the hippocampus (n=6/group, p<0.05, ANOVA). Both changes were attenuated by propofol (n=6/group, respectively p<0,05, ANOVA). ECS under propofol and pentobarbital sodium induced shorter seizures (n=6/group, p<0.05 each, ANOVA).	[10]
**11**	CUMS<br><br>China	Stressors were applied randomly. Stressors included: food or water deprivation for 24 h; cage tilting at 45° for 24 h; footplate stimulation with electrical current (28 V, 1 s/time, 30 times); damping pads for 24 h; continuous lighting for 24 h; cold water swimming at 4°C	Sprague-Dawley<br><br>Male, 6-8 weeks	Administration with a YSD-4G electroconvulsive machine. Electrode placement not reported. Parameter details: sine wave pulses, 0.05 A current, 0.7 ms pulse width, 50 Hz frequency and 1 s duration. Anaesthesia: i.p. injection of dexmedetomidine (DEX, 4, 10, or 25 µg/kg).	SPT, MWM	ECS increased sucrose preference in the SPT (n=7–8, p<0.05, one-way ANOVA). Application of DEX did not significantly affect the ECS-efficacy as measured in the SPT (n=7-8, p>0.05, one-way ANOVA). ECS increased the EL in the MWM (n=8-16, p<0.05, one-way ANOVA), which was ameliorated by DEX (n=8-16, p<0.05, one-way ANOVA).<br>ECS induced the hippocampal expression of NR2B (n=3, p<0.05, one-way ANOVA), which was significantly reversed by the application of 4 µg/kg DEX (n=3, p<0.05, one-way ANOVA). Application of DEX had no significant effect on p-CREB- and BDNF-expression in the hippocampus (n=3, p>0.05, one-way ANOVA).	[11]

		for 5 min; tail squeeze for 1 min.<br><br>Daily for 6 weeks.		Daily for 7 days.			
**12**	CUMS<br><br>China	Stressors were applied randomly. Stressors included: footplate stimulation with electrical current (1 mA, 10 times/min) for 20 min; cold water swimming at 4°C for 5 min; shaking the cage (1 time/s) for 15 min; tail squeeze for 1 min; heat stress (45°C) for 5 min; food deprivation for 48 h; water deprivation for 24 h; wet bedding for 12 h.<br><br>Daily for 21 days.	Sprague-Dawley<br><br>Male, adult (specific age not reported)	Administration with a DX-IIA pulse generator. Electrode placement not reported. Parameter details: 0.05 A current, 0.7 ms pulse width, 50 Hz frequency and 1 s duration. A tonic-clonic seizure with a duration longer than 20 s was used as an ECS success marker. Anaesthesia: i.p. injection of propofol (100 mg/kg).<br><br>Every other day for 14 days.	MWM, OF	In the OF, the behaviour levels of rats in the ECS group and ECS+propofol were higher than the depression-without-treatment group (n=12/group, horizontal level: $p<0.01$ each, vertical level: $p<0.01$ each, ANOVA followed by SNK-q test). There was no significant difference between the ECS group and the ECS+propofol group (n=12/group, horizontal level: $p= 0.485$; vertical level, $p =0.606$, ANOVA followed by SNK-q test). In the MWM, the EL in the ECT group was longer than that in the depression-without-treatment group (n=12/group, $p<0.001$, ANOVA followed by SNK-q test). EL was shorter in the ECT+propofol group compared to the depression-without-treatment group and the ECS group (n=12/group, $p<0.005$ and $p<0.001$ respectively, ANOVA followed by SNK-q test). The depression-without treatment group had a lower swimming time percentage than the ECS+propofol group (n=12/group, $p<0.001$, ANOVA followed by SNK-q test). The ECS+propofol group had a higher swimming time percentage than the ECS group (n=12/group, $p<0.001$, ANOVA followed by SNK-q test). There was no significant difference in swimming time percentage between the ECS group and the depression-without-treatment group (n=12/group, $p>0.05$, ANOVA followed by SNK-q test). The ECS group had a lower concentration of glutamate in the hippocampus compared to depression-without treatment group and the ECS+propofol group (n=12/group, all $p<0.001$, ANOVA followed by SNK-q test). The ECS+propofol rats had a lower concentration of glutamate than the depression-without-treatment group (n=12/group, $p<0.001$, ANOVA followed by SNK-q test). The expression of hippocampal NMDA-NR2B in the ECS+propofol group was lower than in the ECT group (n=12/group, $p<0.001$ for CA1 and CA3 area, respectively, ANOVA followed by SNK-q test).	[12]
**13**	CUMS<br><br>China	Stressors were applied randomly. Stressors included: cold water swimming at 4°C for 5 min; hot water swimming at 45°C for 5 min; tail pinching for 1 min; water deprivation for 24 h; inversion	Sprague-Dawley<br><br>Male, 2–3 months	Administration via bilateral ear clip electrodes with a Niviqure ECS system. Parameter details: bidirectional square wave pulses, 0.8 A amplitude, 1.5 ms width, 125 Hz frequency, 0.8 s duration and 120 mC	SPT, MWM, OF	ECS induced the expression of cannabinoid receptor type 1 (CB1R) (n=at least 3/group, $p<0.029$, independent-samples t-test) in the hippocampus. Following ECS, rats displayed spatial learning and memory impairment with prolonged EL and decreased SET in the MWM (n=9/group, $p<0.001$ each, ANOVA with repeated measures). These impairments were attenuated by CB1R antagonist AM251 (n=9/group, $p<0.001$ each, ANOVA with repeated measures). ECS increased SPP in the SPT (n=6/group, $p<0.001$, ANOVA with repeated measures). ECS increased the total distance travelled and the time spent in the centre square of the OF (n=6/group, all $p<0.001$, ANOVA with repeated measures).	[13]

		of day/night light cycle; food deprivation for 24 h; water deprivation for 24 h; damp sawdust for 24 h.<br><br>Daily for 28 days.		charge. Sham ECS was conducted procedurally similar, yet without current administration. No anaesthesia reported.<br><br>Daily for 7 days.		These effects were not significantly affected by CB1R inhibition (n=6/group, all p>0.05, ANOVA with repeated measures). ECS decreased hippocampal LTP (n=6/group, p<0.001, ANOVA with repeated measures). The reduction in LTP was partially reversed by CB1R antagonist AM251 (n=6/group, p<0.001, ANOVA with repeated measures).	
**14**	CUMS<br><br>China	Stressors were applied randomly. Stressors included: food deprivation for 24 h; water deprivation for 24 h; wet bedding for 12 h; constraint for 2 h; continuous lighting for 24 h; absence of light for 24 h; cage tilting (45°) for 24 h; cold water swimming at 4°C for 5 min; heat stress at 45°C for 5 minutes; tail suspension for 5 min.<br><br>Daily for 28 days.	Sprague-Dawley<br><br>Male, adult (specific age not reported)	Administration via bilateral ear clip electrodes with a Niviqure ECS system. Parameter details: bidirectional square wave pulses, 0.8 A amplitude, 1.5 ms width, 125 Hz frequency, 0.8 s duration and 120 mC charge. Sham ECS was conducted procedurally similar, yet without current administration. All rats treated with ECS showed tonic-clonic seizures with a duration of 10-20 s. Anaesthesia: i.p. injection of propofol (80 mg/kg).<br><br>Daily for 7 days.	SPT,<br>OF	In the SPT, ECS increased SPP compared to sham treatment (n=20/group, p<0.001, two-way repeated-measures ANOVA followed by post hoc analysis). In the OF, ECS increased the total distance travelled and the number of rearing scores, compared to sham treatment (n=20/group, respectively p<0.001, two-way repeated-measures ANOVA followed by post hoc analysis). The fEPSP slope of the sham group was significantly lower compared to the ECS group (n=4/group, p=0.038, two-way repeated-measures ANOVA followed by post hoc analysis). The ECS group showed a higher mean density of SYP in the hippocampal CA3 region than in the sham treatment group (n=8/group, p<0.001, one-way ANOVA followed by post hoc analysis). Hippocampal SYP mRNA levels in the ECS group were higher than in sham treatment group (n=8/group, p<0.001, one-way ANOVA followed by post hoc analysis).	[14]
**15**	CUMS<br><br>China	Stressors were applied randomly. Stressors included: feeding of foreign matter; cold water swimming at 4°C for 5 min; cage tilting at 45° for 24 h; wet caging for 24 h; food deprivation for 24 h; water deprivation for 24 h; tail pinching for 1 min.<br><br>Daily for 21 days.	Sprague-Dawley<br><br>Male, 2-3 months	Administration via ear clip electrodes. Parameter details: 0.8 A amplitude, 1.5 ms width, 120-125 Hz frequency, 0.5 s duration, 55-60 mA current. Sham ECS was conducted procedurally similar, yet without current administration. No anaesthesia reported.<br><br>Daily for 3 days.	SPT,<br>MWM<br>(SPT was performed in all rats prior and after conducting the CUMS model, but not after ECS).	ECS increased EL in the MWM (n= 6/group, p=0.042, one-way ANOVA, followed by the Bonferroni post hoc test). Application of (2R,6R)-HNK+ECS reduced the EL compared to ECS alone (n=6/group, p<0.005, one-way ANOVA, followed by the Bonferroni post hoc test). ECS enhanced autophagy by decreasing the p62 expression level, and increasing the LC3-II/LC3-I ratio and Beclin-1 expression level (n=3/group, all p<0.001, one-way ANOVA, followed by the Bonferroni post hoc test). (2R,6R)-HNK attenuated ECS-induced autophagy by increasing the p62 expression level and lowering the LC3-II/LC3- I ratio and Beclin-1 expression level (n=3, all p<0.001, one-way ANOVA, followed by the Bonferroni post hoc test).	[15]

**16**	CUMS<br><br>China	Stressors were applied randomly. Stressors included: cold water swimming at -4°C for 5min; hot water swimming at 45°C for 5 min; tail suspension for 1 min; continuous lighting for 24 h; food deprivation for 24 h; water deprivation for 24 h; wet bedding for 24 h.<br><br>Daily for 28 days.	Sprague-Dawley<br><br>Male, 2-3 months	Administration via bilateral ear clip electrodes with a Niviqure ECS system. Parameter details: bidirectional square wave pulses, 0.8 A amplitude, 1.5 ms width, 125 Hz frequency, 0.8 s duration and 120 mC charge. Sham ECS was conducted procedurally similar, yet without current administration. No anaesthesia reported.<br><br>Daily for 7 days.	SPT,<br>MWM,<br>OF,<br>Forced swimming test (FST)	In the SPT, ECS increased SPP in rats with SPP< 65% compared to sham (n=10/group, p<0.05, ANOVA followed by Fisher's least significant difference (LSD) test). In the MWM, ECS increased EL and reduced spatial SET compared to sham treatment (n=10/group, p<0.05 respectively, ANOVA followed by Fisher's LSD test). On the 14th day following ECS treatment, there was no significant difference between the sham group and the ECS group in EL and SET (n=10/group, p>0.05 respectively, ANOVA followed by Fisher's LSD test). The activation of A2 subtype astrocytes in rat hippocampal CA1 region reached its maximum on the third day following ECS (n=5/group, p<0.0001, ANOVA followed by Fisher's LSD test). PK2 protein expression in the hippocampal CA1 region was also the highest three days after ECS (n=5/group, p<0.05, ANOVA followed by Fisher's LSD test). After administration of PK2 inhibitor PKRA7, SPP in the SPT was elevated in the ECS+PKRA7 group compared to sham treatment (n=10/group, p<0.01, ANOVA followed by Fisher's LSD test). In the OF, the horizontal grid penetrations and rearing numbers in the ECS (p<0.001 and p<0.0001 respectively) and PKRA7 + ECS groups (p<0.0001 respectively) were higher compared to sham treatment (n=10/group, ANOVA followed by Fisher's LSD test). In the FST, immobility time in the ECS and PKRA7 + ECS groups was shorter compared to sham treatment (n=10/group, p<0.01, ANOVA followed by Fisher's LSD test). In the MWM, SET in the PKRA7+ECS group was shorter and EL was longer compared to the ECS group (n=10/group, p<0.0001 respectively, ANOVA followed by Fisher's LSD test). With PKRA7 treatment, ECS-induced hippocampal A2 subtype was partially reversed (n=5/group, p<0.05, ANOVA followed by Fisher's LSD test).	[16]
**17**	CUMS<br><br>China	Stressors were applied randomly. Stressors included: water deprivation for 24 h; food deprivation for 24 h; shaking for 15 minutes (1 shaking/s); cold water swimming at 4°C for 5 min; tail pinching for 1 min; continuous lighting for 24 h; pad wetting for 24 h; social crowding for 24 h; cage tilting to 45° for 24 h.<br><br>Daily for 28 days.	Sprague-Dawley<br><br>Male, 2-3 months	Administration via bilateral ear clip electrodes with a Niviqure ECS system. Parameter details: bidirectional square wave pulses, 0.8 A amplitude, 1.5 ms width, 125 Hz frequency, 0.8 s duration and 120 mC charge. For sham ECS, bitemporal electrode placement without current administration was used.<br>A tonic-clonic seizure was used as an ECS success marker.	SPT,<br>MWM,<br>OF	In the SPT, the SPP of ECS group and ECS+propofol group was higher than compared to the depression group and propofol group (n=10/group, all p<0.001, repeated-measures ANOVA followed by SNK-q test). There was no significant difference between the ECS group and ECS+propofol group (n=10/group, p=0.794, repeated-measures ANOVA followed by SNK-q test). In the OF, the total distance travelled and incidences of rearing of the rats in the ECS group and ECS+propofol group were higher than those of the rats in the depression group and propofol group (n=10/group, total distance travelled, all p<0.001; incidences of rearing, all p<0.001, repeated-measures ANOVA followed by SNK-q test). There was no significant difference between the ECS group and ECS+propofol group (n=10/group, total distance traveled, p=0.991; incidences of rearing, p=0.455, repeated-measures ANOVA followed by SNK-q test). In the MWM, ECS increased the EL and reduced the SET compared to depression group (n=10/group, p<0.001 respectively, repeated-measures ANOVA followed by SNK-q test). Compared with the ECS group, the ECS+propofol group exhibited shorter EL and longer SET (n=10/group, p<0.001 respectively, repeated-measures ANOVA	[17]

				Anaesthesia: i.p. injection of propofol (80 mg/kg).<br><br>Daily for 7 days.		followed by SNK-q test). The ECS group exhibited lower expression of CaMKIIa and pCaMKIIa in the hippocampal CA1 and CA3 regions (n=10/group, p<0.001 respectively, ANOVA followed by SNK-q test). Compared with ECS group, the ECS+propofol group exhibited higher expression of CaMKIIa and pCaMKIIa in the same hippocampal areas (n=10/group, p<0.001 respectively, ANOVA followed by SNK-q test).	
**18**	CUMS<br><br>Israel	Stressors were applied randomly. Stressors included: cage tilting at 45°C for 7 or 17 h; stroboscopic light for 7 or 17 h; white noise (80 dB) for 3 or 7 h; water soiled cages for 17 h; water deprivation for 20 h; exposure to an empty water bottle for 1 h after water deprivation; food deprivation for 24 h; food and water deprivation for 20 h; paired housing for 7 h; overnight lighting.<br><br>Daily for 28 days.	Sprague-Dawley<br><br>Male, 60 days	Administration via ear clip electrodes with a Siemens Konvulsator 2077 S system. Parameter details: 50 Hz frequency, 1.5 s duration, 100 V voltage. Sham ECS was conducted procedurally similar, yet without current administration. A tonic-clonic seizure with a duration of at least 10 s was used as an ECS success marker. Anaesthesia: i.p. injection of ketamine (85 mg/kg) and promace (0.85 mg/kg)<br><br>Daily for 10 days.	SPT, MWM, FST, Home cage locomotion (HCL), exploration and novelty-induced behaviour	ECS significantly increased SPP in the CUMS animals compared to the CUMS + sham group (n=10-14/group, p<0.05, one-way ANOVA followed by Dunnet's post hoc test). Subconvulsive ES (SCES) of the vPLC and NAC (respectively p<0.05) but not the dorsal PLC (p>0.05) significantly increased SPP relative to the sham-treated CUMS control subjects (n=10-14/group, one-way ANOVA followed by Dunnet's post hoc test). The EL of ECS treated animals was significantly increased and their time in the MWM platform area was significantly reduced relative to the sham-stimulated CUMS group (n=10-14/group, p<0.05 respectively, repeated-measures ANOVA followed by Dunnet's post hoc test). SCES did not show significant differences in the EL and time spent in platform area (n=10-14/group, p>0.05, repeated-measures ANOVA followed by Dunnet's post hoc test). In exploration and novelty-induced behaviour, ECS and SCES did not significantly change the total distance travelled and the number of rearings compared to the respective sham treatment (n=10-14/group, p>0.05, one-way ANOVA followed by Dunnet's post hoc test). The CUMS protocol did not alter home-cage locomotion and did not change the FST performance. Moreover, neither ECT nor SCES treatments affected these behaviours. ECS and SCES of the vPLC and NAC induced a significant increase in dorsal hippocampal BDNF levels compared to sham treatment (n=10-14/group, respectively p<0.05, one-way ANOVA followed by Dunnet's post hoc test).	[18]
**19**	CUMS<br><br>China	Stressors were applied randomly. Stressors included: water deprivation for 24 h; food deprivation for 24 h; damp sawdust for 24 h of continuous lighting; cold water swimming at 4°C for 5 min; shaking for 20 min (1 shaking/s); tail pinching for 1 min; cage tilting at 45°	Sprague-Dawley<br><br>Male, 2-3 months	Administration via ear clip electrodes with a Niviqure ECS system. Parameter details: bidirectional square wave pulses, 0.8 A amplitude, 1.5 ms width, 125 Hz frequency. 0.4, 0.8, 1.2 and 1.6 s duration for 60, 120, 180, and 240 mC charge respectively. Duration and charge varied among the experimental groups	SPT, MWM, OF	The SPP after modified ECS (MECS, ECS following treatment with ketamine and propofol) at different charges increased compared with the respective groups before ECS treatments (n=8/group, all p<0.05, one-way ANOVA with SNK test). Following the ECS treatment at different charges, the number of crossings and rearing in the OF increased compared with the respective groups before the MECS treatment (n=8/group, all p<0.05, one-way ANOVA with SNK test). Following the ECS treatment, EL was shorter and SET was longer for the M120 group in the MWM, compared with the other groups (n=8/group, all p<0.05, one-way ANOVA with SNK test). After MECS treatment, EL was shorter and SET longer compared to the CUMS+saline control group (n=8/group, all p<0.05, one-way ANOVA with SNK test). MECS did not exhibit significant effects on the hippocampal expression of CaMKIIα (n=8/group, p=0,799, one-way ANOVA with SNK test). Compared with the other ECS groups, pCaMKIIα expression significantly increased in the M120 group (n=8/group, p<0.01,	[19]

		for 24 h; social crowding for 24 h.<br><br>Daily for 28 days.		(M60, M120, M180, M240).<br>CUMS rats were used to estimate seizure threshold in ECS under anaesthesia.<br>Sham ECS (group M0) was conducted procedurally similar, yet without current administration.<br>Oxygen was provided and oxygen saturation was monitored during ECS. Rats with oxygen saturation lower than 95% were excluded.<br>Anaesthesia: i.p. injection of ketamine (10 mg/kg) and propofol (80 mg/kg) or saline (8 ml/kg).<br><br>Daily for 7 days.		one-way ANOVA with SNK test). The expression of pGluR1 was the highest in M120 group, compared with all other groups (n=8/group, p<0.01, one-way ANOVA with SNK test). The M120 group exhibited a significantly increased level of pCREB in the hippocampus, compared with the other ECS groups (n=8/group, p<0.01, one-way ANOVA with SNK test).	
**20**	CUMS<br><br>China	Stressors were applied randomly. Stressors included: cold water swimming at 4°C for 5 min; tail pinching for 1 min; food deprivation for 24 h; water deprivation for 24 h; shaking for 20 min (1 shake/s); social crowding with the cage being tilted (30°) for 24 h; continuous lighting for 24 h; housing in a soiled cage for 24 h; heat stress (45°C) for 5 min; undesirable confinement for 2 h.<br><br>Daily for 28 days.	Wistar<br><br>Male, adult (specific age not reported)	Administration via bilateral ear clip electrodes with a Niviqure ECS system. Parameter details: bidirectional square wave pulses, 0.8 A amplitude, 1.5 ms width, 125 Hz frequency. 0.4, 0.8, 1.2 and 1.6 s duration for 60, 120, 180, and 240 mC charge respectively. Duration and charge varied among the experimental groups.<br>CUMS rats were used to estimate seizure threshold in ECS under anaesthesia.<br>Sham ECS was conducted procedurally similar, yet without current administration.	SPT, OF	Propofol reduced the SPP at low ECS charge (60 mC, n=8/group, p<0.01, ANOVA followed by Bonferroni's post-hoc test) and enhanced SPP at medium or high ECS charges (at and above 120 mC, n=8/group, p<0.05, ANOVA followed by Bonferroni's post-hoc test) in the SPT compared to saline treatment. In the OF, there was no significant difference between the horizontal ambulation of propofol and saline groups at any charges except for 60 mC (the value for saline-treatment at 60 mC was larger than propofol-treatment at 60 mC, p<0.01, for other groups p>0.05, n=8/group, ANOVA followed by Bonferroni's post-hoc test). Propofol reduced the rearing number in the OF at 60 mC and 120 mC, (n=8/group, p<0.05, ANOVA followed by Bonferroni's post-hoc test), but there was no significant difference at other charges (n=8/group, p>0.05, ANOVA followed by Bonferroni's post-hoc test). Propofol weakened the ECS-induced elevation of hippocampal BDNF levels at a lower charge (60 mC, n=6/group, p<0.05, ANOVA followed by Bonferroni's post-hoc test), but it enhanced the ECS-induced elevation of BDNF level at higher charges (120 mC and 180 mC, n=6, p<0.05, ANOVA followed by Bonferroni's post-hoc test), as compared to saline. The enhancement was not significant at the highest charge (240 mC, n=6/group, p>0.05, ANOVA followed by Bonferroni's post-hoc test).	[20]

				Oxygen was provided and oxygen saturation was monitored during ECS. Rats with oxygen saturation lower than 95% were excluded.<br>Anaesthesia: i.p. injection of propofol (90 mg/kg) or saline (9 ml/kg).<br><br>Daily for 7 days.			
**21**	CUMS<br><br>China	Stressors were applied randomly.<br>Stressors included: cold water swimming at 4°C for 5 min; tail pinching for 1 min; food deprivation for 24 h; water deprivation for 24 h; social crowding in tilted cage (30°) for 24 h; shaking for 20 min (1 shake/s); continuous lighting for 24 h; housing in a soiled cage for 24 h; heat stress at 45°C for 5 min; undesirable confinement for 2 h.<br><br>Daily for 28 days.	Wistar<br><br>Male, adult (specific age not reported)	Administration via ear clip electrodes with a Niviqure ECS system.<br>Parameter details: bidirectional square wave pulses, 0.8 A amplitude, 1.5 ms width, 125 Hz frequency, 0.8 s duration and 120 mC charge.<br>Sham ECS was conducted procedurally similar, yet without current administration.<br>Oxygen was provided and oxygen saturation was monitored during ECS.<br>Anaesthesia: i.p. injection of propofol (90 mg/kg) or saline (9 ml/kg).<br><br>Daily for 7 days.	SPT,<br>MWM,<br>OF	ECS with propofol or saline increased the SPP in the SPT (n=10/group, each p<0.001 compared to sham, ANOVA followed by Fisher's LSD test) and increased the horizontal ambulation distance and the number of rearing events in the OF (n=10/group, all p<0.001 compared to sham, ANOVA followed by Fisher's LSD test). Administration of propofol shortened the EL in the MWM after ECS, compared to ECS+saline (n=10/group, p<0.003, ANOVA, followed by Fisher's LSD test). ECS+Propofol elevated the hippocampal expression of PSD-95 (n=6/group, p<0.004, ANOVA followed by Fisher's LSD test), CREB and pCREB (n=5-6/group, p<0.001 respectively, ANOVA followed by Fisher's LSD test) in comparison to the ECS+saline group.	[21]
**22**	CUMS<br><br>Germany	Stressors were applied randomly.<br>Stressors included: cold water swimming at 15°C for 5-10 min; hot water swimming at 40°C for 5-10 min; food deprivation for 21.5-24 h; water deprivation for 14-21 h; continuous	Wistar outbred<br><br>Male, 7-8 weeks	Administration of auricular ECS via ear clip electrodes with an Ugo Basile ECS 57800 Unit.<br>Parameter details for auricular ECS: bidirectional square wave pulses, 0.9 ms width, 100 Hz frequency, 0.5 s	Sucrose consumption test (SCT),<br>OF,<br>FST,<br>novelty- induced hypophagia test (NIHP),<br>social interaction test (SIT)	Cortical ECS increased the sucrose consumption of CUMS rats (n=9 for CUMS+cortical ECS vs n=29 for CUMS before ECS, p=0.0427). Auricular ECS did not significantly change the sucrose consumption (n=10 for CUMS+auricular ECS vs n=29 for CUMS before ECS, p>0.05). Cortical (n=10 for cortical ECS vs 9 for sham ECS, p<0.01) but not auricular ECS (n=10 for auricular ECS vs 9 for sham ECS, p>0.05) compared to sham ECS decreased the duration of immobility in the FST. Auricular ECS exerted increased latency to eat and reduced food intake in the NIHP compared to sham ECS or cortical ECS (n= 10 for auricular ECS vs 9 for sham ECS and n=10 for cortical ECS, all p<0.01). No significant differences between sham ECS (n=9), cortical ECS	[22]

		lighting for 36 h; social crowding for 7 h; wet bedding for 16 h; restraint stress for 0.5-1 h.<br><br>Daily for 21 days.		duration and 6.75-7.65 mC charge. Administration of cortical ECS via bifrontal screw electrodes with the A310 Accupulser system. Parameter details for cortical ECS: bidirectional square wave pulses, 1 ms width, 100 Hz frequency, 1 s duration and 1.3-1.8 mC charge. Sham auricular and sham cortical ECS was conducted procedurally similar, yet without current administration. No anaesthesia reported.<br><br>Daily for 5 days. The CUMS model was continued during the ECS-treatment period. Two further ECS sessions were scheduled during CUMS after completion of the behavioural tests and before decapitation.		(n=10) and auricular ECS (n=10) were observed in the OF (time in centre, p=0.317) and SIT (distance between animals, p=0.981; time in body contact, p= 0.613). P11 promoter methylation (p=0.017, mixed linear model for repeated measurements) and p11 mRNA expression (p<0.001 comparing positive responders with anhedonic-like sham rats and p=0.004 comparing positive responders and non/negative responders, mixed linear models for repeated measurements followed by Sidak's posthoc test) was significantly increased in the PFC of ECS responders (n=5) compared to anhedonic-like sham rats (n=6) and rats with no, or negative response to ECS in the SCT (n=10). ECS responders (n=5) and non/negative ECS responders (n=10) in SCT exhibited higher BDNF protein expression than anhedonic-like sham rats (n=6) (p<0.05). When not otherwise mentioned, one-way ANOVA or Kruskal-Wallis test with posthoc Dunn's or Dunnett's multiple comparison tests were used for statistical analysis.<br><br>P11 promoter methylation was higher in patients with pharmacoresistant MDD who responded to ECT than in non-responders before each ECT (n=4 for responders vs n=6 for non-responders, p<0.0001, derived from a mixed linear model for repeated measurements including covariates age and sex). ECT responders in an independent sample showed a higher baseline p11 promoter methylation compared to non-responders (n=50 for responders vs n=15 for non-responders, p=0.014, derived from mixed linear modelling including the covariates age and sex).	
**23**	CUMS<br><br>China	Stressors were applied randomly. Stressors included: cold water swimming at 4°C for 5 min; hot water swimming at 45°C for 5 min; food deprivation for 24 h; water deprivation for 24 h; continuous lighting for 24 h; tail pinching for 1 min; shacking for 20 min; damp sawdust	Sprague-Dawley<br><br>Male, 2-3 months	Administration via bilateral ear clip electrodes with a Niviqure ECS system. Parameter details: bidirectional square wave pulses, 0.8 A amplitude, 1.5 ms width, 125 Hz frequency, 0.8 s duration and 120 mC charge. Sham ECS was conducted procedurally similar, yet without current administration.	SPT, MWM	CUMS+ECS rats demonstrated sucrose preference similar to control animals and significantly higher than that of CUMS-only rats (n=8/group, p<0.05, one-way ANOVA with Bonferroni correction and post hoc test). CUMS+ECS rats displayed spatial learning and memory impairment with higher EL and shorter SET (n=8/group, p<0.005, each compared CUMS-only animals, repeated-measures ANOVA and one-way ANOVA with Bonferroni correction and post hoc test, respectively) in the MWM. Compared to CUMS-only rats, CUMS+ECS caused LTP impairment in hippocampal slices (CA1) as well as an increase in long-term depression, depotentiation enhancement, and post-tetanic potentiation impairment (n=8/group, p<0.001 and p=0.025 and p<0.001 and p<0.001, respectively, one-way ANOVA with Bonferroni correction and post hoc test).	[23]

		for 24 h; cages tilting (45°) for 24 h<br><br>Daily for 28 days.		No anaesthesia reported.<br><br>Daily for 7 days.			
**24**	CUMS<br><br>China	Stressors were applied randomly. Stressors included: isolation; food deprivation for 24 h; water deprivation for 24 h; cold water swimming at 4°C for 5 min; hot water swimming at 45°C for 5 min; cages tilting (45°) for 24 h; shaking (1 shake/s) for 15 min; tail pinching for 1 min; soiled litter for 24 h; exposure to a reversed light/dark cycle.<br><br>Daily for 28 days.	Sprague-Dawley<br><br>Male, 2-3 months	Administration via ear clip electrodes with a modified Niviqure ECS system. Parameter details: biphasic square wave pulses, 0.8 A amplitude, 1.5 ms width, 125 Hz frequency and 0.8 s duration. Sham ECS was conducted procedurally similar, yet without current administration. Oxygen was provided and oxygen saturation was monitored during ECS. A tonic-clonic seizure was used as ECS success marker. Anaesthesia: i.p. injection of propofol (80 mg/kg) or saline (8 ml/kg).<br><br>Daily for 7 days.	SPT, OF, MWM	Both in the CUMS+ECS+saline and CUMS+ECS+propofol group, sucrose preference was significantly higher than in control CUMS+sham ECS+saline rats (n=10/group, p<0.001 and p=0.007, repeated-measures ANOVA). Similar statistics were found for the total distance travelled and rearing times in the OF (n=10/group, each p<0.001, repeated-measures ANOVA). In the MWM, EL increased in both the CUMS+ECS+saline and CUMS+ECS+propofol group compared to CUMS+shamECS+saline controls (n=10/group, p<0.001 and p=0.013, repeated-measures ANOVA), while SET decreased (n=10/group, p<0.001 and p=0.032, repeated-measures ANOVA). CUMS+ECS+saline animals showed an increased hippocampal expression of Beclin-1, LC3-II/I, and synaptophysin (n=10/group, p<0.001 and p<0.001 and p<0.001, one-way ANOVA) compared to controls with CUMS+shamECS+saline.	[24]
**25**	CUMS<br><br>China	Stressors were applied randomly. Stressors included: cold water swimming at 4°C for 5 min; hot water swimming at 40°C for 5 min; food deprivation for 24 h; water deprivation for 24 h; cage tilting (45°C) for 24 h; wet bedding for 24 h; shaking for 10 min;	Sprague-Dawley<br><br>Male, 7-8 weeks	Administration via bilateral ear clip electrodes with a Niviqure ECS system. Parameter details: bidirectional square wave pulses, 0.8 A amplitude, 1.5 ms width, 125 Hz frequency, 0.8 s duration and 120 mC charge. Sham ECS was conducted procedurally	SPT, OF, FST, MWM	After CUMS, the rats with SPP lower than 65% were randomized into CUMS+ECS and CUMS+sham ECS groups. In the OF, CUMS+ECS rats showed a higher number of squares crossed compared to the CUMS+sham ECS group (n=5/group, p<0.05, t-test). In the FST, CUMS+ECS rats showed a reduced immobility time compared to CUMS+sham ECS rats (n=5/group, p<0.05, t-test). In the MWM, the EL of rats in CUMS+ECS group was increased and their SET was reduced compared to the CUMS+sham ECS group (n=5/group, p<0.05 respectively, t-test). In the hippocampal CA1 region, the levels of IL-1β, IL-18 and the NLRP3 expression level were higher in the CUMS+ECS group compared with the CUMS+sham ECS group (n=5/group, p<0.01 and p<0.05 and p<0.05 respectively, t-test).	[25]

	tail suspension for 1 min.<br><br>Daily for 28 days.	similar, yet without current administration. No anaesthesia reported.<br><br>Daily for 7 days.	Following CUMS, the rats with SPP lower than 65% were randomized into CUMS+ECS, CUMS+sham ECS, CUMS+ECS+MCC950 (NLRP3 inhibitor) and CUMS+sham ECS+MCC950 groups.<br>In the SPT, SPP was higher in the CUMS+ECS and CUMS+ECS+MCC950 group compared to CUMS+sham ECS (n=5/group, p<0.05 each, one-way repeated-measures ANOVA with Fisher's LSD test). In the OF, the number of squares crossed in CUMS+MCC950+ECS increased compared to the CUMS+sham ECS group and decreased compared to the CUMS+ECS group (n=5/group, all p<0.05, one-way repeated measures ANOVA with Fisher's LSD test). In the FST, CUMS+ECS and CUMS+ECS+MCC950 rats showed a reduced immobility time compared to CUMS+sham ECS animals (n=5/group, p<0.05 each, one-way repeated-measures ANOVA with Fisher's LSD test). In the MWM, the CUMS+ECS+MCC950 rats showed a shorter EL and a longer SET compared to CUMS+ECS rats (n=5/group, all p<0.05, one-way repeated-measures ANOVA with Fisher's LSD test). The SET of CUMS+ECS+MCC950 rats was shorter compared to CUMS+sham ECS (n=5/group, p<0.05, one-way repeated-measures ANOVA with Fisher's LSD test).<br>The CUMS+ECS+MCC950 group showed a reduced expression of IL-1β and IL-18 in the hippocampal CA1 region compared to CUMS+ECS group (n=5/group, p<0.05 each, one-way repeated-measures ANOVA with Fisher's LSD test). CUMS+ECS rats showed a higher expression of Caspace-1 and ASC in the hippocampal CA1 region compared to CUMS+sham ECS (n=5/group, p<0.05 each, one-way repeated-measures ANOVA with Fisher's LSD test). MCC950 partially reversed the reduced expression of NLRP3, Caspace-1 and ASC after ECS (n=5/group, p<0.05 each, one-way repeated-measures ANOVA with Fisher's LSD test).<br>CUMS+ECS rats showed higher microglial activation in the hippocampal CA1 region compared to CUMS+sham ECS rats (n=5/group, p<0.05, one-way repeated-measures ANOVA with Fisher's LSD test). The microglial activation in the hippocampal CA1 region of CUMS+MCC950 rats was reduced compared to CUMS+ECS rats (n=5/group, p<0.05, one-way repeated-measures ANOVA with Fisher's LSD test).<br>The CUMS+ECS+MCC950 group showed an increased number of synapses and an increased postsynaptic density compared to CUMS+ECS (n=5/group, p<0.05 respectively, one-way repeated-measures ANOVA with Fisher's LSD test).<br>PSD-95 protein expression in the hippocampal CA1 region was lower in the CUMS+ECS group compared to the CUMS+sham ECS group (n=5/group, p<0.01, one-way repeated-measures ANOVA with Fisher's LSD test). The protein expression of PSD-95 in the CA1 region was increased in the CUMS+MCC950+ECS

						group compared to the CUMS+ECS group (n=5/group, p<0.05, one-way repeated-measures ANOVA with Fisher's LSD test).	
**26**	CUMS<br><br>Israel	Stressors were applied randomly. Stressors included: water deprivation for 12h during the dark period; cage shaking with music and lights on for 1 h; continuous lighting for 12 h over night; lights-off for 3h during daylight; flashing light for 3h; placement in a cold room; mild restraint for 2h, cage tilt (45°) for 14h; wet sawdust for 14h; exposure to fox, ferret, bobcat, coyote or rat odour for 1 h; noise in the room for 3h.<br><br>Daily for 35 days.	C57BL/6J. Half of the mice received a special diet (PLX5562) to induce microglia depletion.<br><br>Male, 4-6 months	Administration via ear clip electrodes with an Ugo Basile ECS unit. Parameter details: 18 mA current, 0.5 ms pulse width, 100 Hz frequency and 0.3 s duration.<br>Sham ECS was conducted procedurally similar, yet without current administration.<br>Anaesthesia: administration of isoflurane, no further details reported.<br><br>Three times per week for 2.5 weeks for a total of 8 ECS sessions.	SPT,<br>FST,<br>social exploration	ECS ameliorated the effects of CUS by increasing the SPP in the SPT (n=6-7/group, p<0.001, analysis of variance (ANOVA)) and by reducing immobility time in the FST (n=6-11/group, p<0.01, ANOVA). ECS did not significantly change the effects of CUMS in the social exploration test (n=6-7/group, p>0.05, ANOVA). ECS increased hippocampal neurogenesis in CUMS-exposed mice by increasing the number of doublecortin (DCX)-positive neurons in the dentate gyrus (DG) compared to sham (n=3-6/group, p<0.005, ANOVA). Microglia depletion abrogated the therapeutic effects of ECS in CUMS mice by reducing the SPP in the SPT (n=6-7/group, p<0.05, ANOVA) and increasing the immobility time in the FST (n=6-11/group, p<0.001, ANOVA). ECS after microglia depletion reduced the number of doublecortin-positive neurons in the DG compared to sham (n=3-6/group, p<0.005, ANOVA). ECS significantly increased the expression of hippocampal genes associated with neurogenesis and synaptic neurotransmission and reduced expression of immune checkpoint genes compared to sham treatment (p<0.05, RNA-seq analysis validated by qPCR, raw p-values were adjusted using Benjamini and Hochberg's procedure).	[26]
**27**	Chronic restraint stress (CRS)<br><br>Denmark	Placement into flexible wire mesh restrainers for 6 h.<br><br>Daily for 21 days.	Wistar<br><br>Male, 8 weeks	Administration transcranially with a PSCC-10 pulse-stimulator. Electrode placement not reported. Parameter details: square wave pulses, 50 mA current, 50 Hz frequency, 0.5 s duration.<br>Sham ECS was conducted procedurally similar, yet without current administration.<br>A tonic-clonic seizure with a duration of 20-30	FST	There was no significant difference in the immobility time between sham-ECS (controls without CRS) and CRS+ECS treated rats in the FST (n=12/group, p>0.05, two tailed Student's t test). The total number of BrdU-positive neurons was increased in rats subjected to CRS+ECS at 3 (n=11-12/group, p<0.001, two-way ANOVA followed by Tukey's post-hoc t tests), 6 (n=11-12/group, p<0.05, two-way ANOVA followed by Tukey's post-hoc t tests), and 12 (n=11-12/group, p<0.05, two-way ANOVA followed by Tukey's post-hoc t tests) months following the procedure, compared to controls with sham ECS+no CRS. There was no significant difference in the dentate gyrus granule cell layer (GCL) volume/brain weight ratios in control animals and CRS+ECS treated rats (n=11-12/group, p>0.05, two-way ANOVA followed by Tukey's post-hoc t tests). ECS treatment did not predict the outcome of the FST (mediated analysis, direct effect: c=-0.39, p=0.7205).	[27]

				s was used as an ECS success marker.<br>No anaesthesia reported.<br><br>Three days a week for 21 days.			
**28**	CRS<br><br>Denmark	Placement into flexible wire mesh restrainers for 6 h.<br><br>Daily for 21 days.	Wistar<br><br>Male, 7-8 weeks	Administration transcranially via metal forceps with the PSCC-10 pulse-stimulator.<br>Electrode placement not reported.<br>Parameter details: square wave pulses, 50 mA current, 50 Hz frequency, 0.5 s duration.<br>Sham ECS was conducted procedurally similar, yet without current administration.<br>A tonic-clonic seizure with a duration of 20-30 s was used as an ECS success marker.<br>No anaesthesia reported.<br><br>Three days a week for 21 days.	FST	In the FST, CRS rats showed an increase of immobility time compared to the control group (no CRS+sham ECS, n=12 for CRS group vs 11 for control group, $p<0.05$, ANOVA followed by Bonferroni post hoc t tests). The immobility time of CRS+ECS rats was not significantly different compared to the control group (n=11/group, $p>0.05$, ANOVA followed by Bonferroni post hoc t tests).<br>CRS+ECS rats showed a higher number of BrdU-positive neurons in the hippocampal subgranular zone compared to control rats (no CRS+sham ECS, n=11/group, $p<0.01$, Kruskal-Wallis analysis followed by Dunn's multiple comparison tests). There was no significant difference in the total number of neurons and the volume of the hippocampal GCL in CRS+ECS rats compared to control rats (no CRS+sham ECS, n=11/group, $p>0.05$, ANOVA followed by Bonferroni post hoc t tests and Kruskal–Wallis analysis, respectively). CRS rats did not show a significant difference in the number of BdrU-positive neurons in the hippocampal subgranular zone (n=12 for CRS group vs 11 for control group, $p>0.05$, Kruskal-Wallis analysis), in the total number of neurons and the volume of GCL compared to controls (no CRS+sham ECS) rats (n=12 for CRS group and n=11 for control group, $p>0.05$, ANOVA followed by Bonferroni post hoc t tests and Kruskal–Wallis analysis, respectively).	[28]
**29**	CRS<br><br>Denmark	Placement into wire mesh restrainers for 6 h.<br><br>Daily for 21 days.	Sprague-Dawley<br><br>Male, (specific age not reported)	Administration of ECS transauricularly.<br>ECS device not reported.<br>Parameter details: 50 mA current, 50 Hz and 0.5 s duration.<br>Rats treated with ECS displayed a tonic-clonic seizure with a duration of 20-30 s.<br>No anaesthesia reported.<br><br>Three times a week for 21 days.	FST	CRS+ECS treated rats showed significantly increased glucocorticoid receptor mRNA levels in the CA1, CA3, CA3c and DG hippocampal regions compared to CRS rats (n=12/group, $p<0.05$ for CA1, CA3 and CA3c, $p<0.01$ for DG, ANOVA followed by post hoc Student's t test). There was no significant difference in the glucocorticoid receptor mRNA levels in the CA1, CA3, CA3c and DG regions between CRS+ECS treated animals and control animals (n=12/group, all $p>0.05$, ANOVA followed by post hoc Student's t test). CRS+ECS treated animals showed significantly increased mineralocorticoid receptor mRNA levels in the DG compared to control animals (n=12/group, $p<0.05$, ANOVA followed by post hoc Student's t test). There was no significant difference in the mineralocorticoid receptor mRNA levels in the CA1, CA3, CA3c regions between CRS+ECS treated animals and control animals (n=12/group, all $p>0.05$, ANOVA followed by post hoc Student's t test).<br>ECS resulted in decreased immobility time (n=12/group, $p<0.01$, Mann-Whitney test) and increased latency to immobility	[29]

						(n=12/group, p<0.01, Mann-Whitney test) in CRS+ECS rats compared to CRS rats.	
**30**	Chronic water immersion and restraint stress<br><br>Japan	Placement in a restrainer tube with multiple air holes and immersion into water for 3-7 h.<br><br>Daily for 15 days	C57BL/6J<br><br>Male, 7-8 weeks	Administration via ear clip electrodes with an Ugo Basile ECS unit. Parameter details: 30 mA current, 0.5 ms pulse width, 80 Hz frequency and 1.2 s duration. Sham ECS was conducted procedurally similar, yet without current administration. Mice treated with ECS displayed a tonic-clonic seizure with a duration of 10-20 s. Anaesthesia: intraperitoneal (i.p.) injection of pentobarbital (50 mg/kg).<br><br>Once daily for a total of 10 ECS sessions in 14 days.	Measurement of locomotor activity, FST, NSF	There was no significant difference between stressed and control mice without treatment or stress+ECS and stressed mice without treatment in locomotor activity (n=8/group, all p>0.05, two-way ANOVA followed by Tukey post hoc test) and in immobility time in the FST (n=6-8/group, all p>0.05, two-way ANOVA followed by Tukey post hoc test). Latency to feeding in the NSF was higher in stressed mice compared to control mice (n=10 for stress vs 5 for control, p<0.05, two-way ANOVA followed by the Tukey post hoc test). There was no significant difference in latency to feeding between stress+ECS and stressed mice without treatment (n=8 for ECS vs 10 for no treatment, p>0.05, two-way ANOVA followed by Tukey post hoc test). There was no significant difference in the density of BrdU/DCX positive cells in the hippocampal DG between the groups (n=7-9/group, p>0.05, two-way ANOVA followed by the Tukey post hoc test).	[30]
**31**	Chronic social stress (CSS)<br><br>Netherlands / Switzerland	Each CSS mouse was placed in the cage of a CD-1 mouse, separated by a transparent, perforated divider. After that, the two mice were placed in the same compartment for a cumulative total of 60 s of physical attacks or 10 min maximum. Rotation of CSS and CD-1 mice pairings every day.	C57BL/6J<br><br>Male, 10 weeks	Administration via ear clip electrodes with an Ugo Basile ECS unit. Parameter details: 80 mA current, 0.5 ms pulse width, 50 Hz frequency, 1 s duration and 80 mC charge. Sham ECS was conducted procedurally similar, yet without current administration. Mice treated with ECS displayed a tonic-clonic seizure with a duration of 5-20 s (average duration: 13.0±2.2 s).	Tone-shock fear learning and memory, fear conditioning, treadmill fatigue test, hot plate test	CSS did not result in an increased fear conditioning- (n=11-12/group, group effect, p=0.26-0.41, two-way ANOVA) or expression (n=11-12/group, group effect, p=0.62-0.74, two-way ANOVA), but in increased physical fatigue (n=11-12/group, group effect, p<0.03, two-way ANOVA). ECS mice exhibited reduced fear learning (n=11-12/group, treatment effect, p=0.01, two-way ANOVA) and fear memory expression (n=11-12/group, treatment effect, p<0.0001, two-way ANOVA) and showed increased running fatigue in a treadmill test (n=11-12/group, treatment effect, p<0.04, two-way ANOVA) compared to sham-treated animals. No altered pain sensitivity in hot plate test in CSS (n=6/group, p=0.33, ANOVA), nor ECS (n=6/group, p=0.16, two-way ANOVA) was found.<br>ECS showed no effect on microglial activity in CSS mice (n=11-12/group, group effect, p=0.17-0.87, two-way ANOVA). However, ECS mice showed increased microglial activity in hippocampal	[31]

		Daily for 15 days.		Anaesthesia: administration of isoflurane (3% in O_2 at 800 ml/min).<br><br>Daily for 10 days.		regions CA1 and CA3 (n=11-12/group, treatment effect, p=0.01, two-way ANOVA).<br>Reduced hippocampal cholinergic fibre density in the hilus and CA3 region was described after ECS (n=11-12/group, treatment effect, p<0.001, two-way ANOVA).	
**32**	Maternal deprivation early in life<br><br>Brazil	Maternal deprivation by removing the mother from the residence box for 3 h.<br><br>Daily for 10 days (postnatal days 1-10).	Wistar<br><br>Male and female. Begin of treatment at 60th postnatal day.	Administration transcranially with the PSCC-10 pulse-stimulator. Electrode placement not reported. Parameter details: square wave pulses, 50 mA current, 50 Hz frequency, 0.5 s duration.<br>Sham ECS was conducted procedurally similar, yet without current administration.<br>A tonic-clonic seizure with a duration of 20-30 s was used as an ECS success marker.<br>No anaesthesia reported (i.p. injection of ketamine with a dose of 5 mg/kg was regarded as treatment and took place twice a week).<br><br>Three times a week for 21 days.	Splash test,<br>OF,<br>FST	In female deprived rats, ECS did not significantly change immobility time in the FST compared to deprived+saline+sham animals (n=15/group, p>0.05, one-way ANOVA followed by Tukey's post hoc test). In females, there was a decrease in immobility time in the deprived+ECS+escitalopram+ketamine group when compared to deprived+saline+sham (n=15/group, p<0.05, one-way ANOVA followed by Tukey's post hoc test). In females, the treatments with ECS+escitalopram and ECS+escitalopram+ketamine increased grooming time in the splash test when compared to the deprived+saline+sham group (n=15/group, p<0.05, one-way ANOVA followed by Tukey's post hoc test). ECS alone did not significantly change grooming time compared to deprived+saline+sham animals (n=15/group, p>0.05, one-way ANOVA followed by Tukey's post hoc test). In females in the OF, there was no difference between any treatment groups, including ECS, for the number of crossings and rearings compared to deprived+saline+sham (n=15/group, p>0.05, one-way ANOVA followed by Tukey's post hoc test). In females, the treatments with ECS, ECS+escitalopram and ECS+escitalopram+ketamine reduced the TBARS levels in the hippocampus compared to the deprived+saline+sham group (n=15/group, p<0.05, one-way ANOVA followed by Tukey's post hoc test). In females, only ECS treatment increased catalase activity in the PFC (n=15/group, p<0,05, one-way ANOVA followed by Tukey's post hoc test), but not in the hippocampus, compared to deprived+saline+sham (n=15/group, p>0.05, one-way ANOVA followed by Tukey's post hoc test). In male rats, the treatments with ECS, ECS+ketamine, ECS+escitalopram and ECS+escitalopram+ketamine decreased the immobility time in the FST when compared with the deprived+saline+sham group (n=15/group, p<0.05, one-way ANOVA followed by Tukey's post hoc test). In males, ECS, ECS+escitalopram and ECS+ketamine increased grooming time in the splash test compared to deprived+saline+sham (n=15/group, p<0.05, one-way ANOVA followed by Tukey's post hoc test). In males, there was no significant difference, between any treatment group, including ECS, for the number of crossings in the OF (n=15/group, p>0.05, one-way ANOVA followed by Tukey's post hoc test). The number of rearings was reduced in groups treated with ECS, ECS+escitalopram and ECS+escitalopram+ketamine when compared to the deprived+saline+sham group (n=15/group, p<0.05, one-way ANOVA followed by Tukey's post hoc test). In	[32]

						males, the treatments with ECS+escitalopram but not ECS alone (p>0.05) reduced the TBARS levels in the hippocampus compared to the deprived+saline+sham group (n=15/group, p<0.05, one-way ANOVA followed by Tukey's post hoc test). In males, only ECS+escitalopram+ketamine increased the catalase activity in the hippocampus (n=15/group, p<0.05, one-way ANOVA followed by Tukey's post hoc test). ECS did not significantly change the catalase activity in the PFC and in hippocampus (n=15/group, p>0.05, one-way ANOVA followed by Tukey's post hoc test).	
**33**	Surgical model<br><br>China	Olfactory bulbectomy	Sprague-Dawley<br><br>Male, 24 weeks	Administration of ECS via bitemporally placed electrodes with a Harvard-sine wave ECS device.<br>Parameter details: square wave, 50 mA current, 50 Hz frequency, 1 s duration.<br>A tonic-clonic seizure was used as an ECS success marker.<br>No anaesthesia reported.<br><br>Once every two days for a total of 7 ECS sessions.	MWM	ECS treatments resulted in learning and memory impairments, with prolonged EL and a shortened SET in the MWM (n=8/group, p<0.001 and p=0.043 respectively, one-way ANOVA).<br>ECS significantly increased the concentration of Glu in the hippocampus of rats (n=8/group, p<0.001, one-way ANOVA).<br>ECS increased the protein expression levels of p-AT8^{Ser202} and GSK-3β^{1H8} in the hippocampus of rats (n=8/group, respectively p<0.001, one-way ANOVA), whereas 2,6-diisopropylphenol and the NMDA receptor antagonist MK-801 reduced their expression levels (n=8/group, each p<0.001, one-way ANOVA followed by SNK-q-test).	[33]
**34**	Pharmacological model<br><br>Iran	Intraperitoneal injections of low doses reserpine (0.2 mg/kg) or saline.<br><br>Daily for 14 days.	Wistar<br><br>Male, adult (specific age not reported)	Administration of ECS via ear clip electrodes with an Ugo Basile ECT Unit 57800 system.<br>Parameter details: bidirectional square wave pulses, 60 mA amplitude, 0.5 ms width, 100 Hz frequency, 1 s duration.<br>A tonic-clonic seizure with a duration less than 20 s was used as an ECS success marker.<br>No anaesthesia reported.<br><br>Daily for 7 days.	SPT,<br>FST,<br>Y maze test (YMT), novel object recognition test (NORT)	ECS alone did not significantly affect the immobility time of reserpine-treated rats in the FST (n=15/group, p>0.05, two-way ANOVA with post-hoc test). ECS increased the SPP of reserpine-treated rats in the SPT (n=15/group, p<0.05, two-way ANOVA with post-hoc test), but reduced the discrimination index in the NORT and diminished alternation behaviours parameters in the YMT (respectively n=15/group, p<0.05, two-way ANOVA with post-hoc test). Treatment with 20 mg/kg hesperetin/+ECS reduced the immobility time of reserpine-treated rats in the FST and increased their SPP in the SPT (n=15/group, p<0.05, two-way ANOVA with post-hoc test). Treatment with 20 mg/kg hesperetin+ECS increased the discrimination index in the NORT and increased alternation behaviours parameters in YMT in comparison to ECS stimulation of reserpine-treated rats (respectively, n=15/group, p<0.01, two-way ANOVA with post-hoc test). There was a significant decrease of malondialdehyde after treatment with hesperetin (both 10 and 20 mg/kg)+ECS as compared to ECS alone (n=9/group, p<0.001, two-way ANOVA with post-hoc test) and a significant increase of hippocampal BDNF after treatment with ECS and high dose (20 mg/kg)	[34]

						hesperetin as compared to ECS (n=9/group, p<0.001, two-way ANOVA with post-hoc test).	
**35**	Neuro-endocrine model<br><br>Japan	Subcutaneous (s.c.) injection of dexamethason (1 mg/kg).<br><br>Daily for 14 days.	Wistar<br><br>Male, (specific age not reported)	Administration of ECS via ear clip electrodes with an Ugo Basile ECT Unit 7800 system.<br>Parameter details: 60 Hz frequency, 0.5 s duration and 80 mA current.<br>Sham ECS was conducted procedurally similar, yet without current administration.<br>A tonic-clonic seizure was used as an ECS success marker.<br>No anaesthesia reported.<br><br>Daily for 14 days.	Wet-dog shake (WDS) behaviours	The number of DOI-induced WDS behaviours in the Dex+ECS group was reduced in comparison with the Dex+sham group (n=not reported, p<0.01, one-way ANOVA followed by Scheffe's test). The number of 5-HT-2A receptor binding sites in the frontal cortex was not significantly different between the Dex+ECS and Dex+sham groups (n=11-12/group., p>0.05, one-way ANOVA followed by Scheffe's test).	[35]
**36**	Neuro-endocrine model<br><br>Japan	Injection (s.c.) of 100 µg ACTH or saline.<br><br>Daily for 14 days.	Wistar<br><br>Male, 8-10 weeks	Administration of ECS via corneal electrodes. ECS device not reported.<br>Parameter details: 50 mA current, 0.2 s duration and 100 V voltage.<br>Sham ECS was conducted procedurally similar, yet without current administration.<br>All rats treated with ECS displayed a tonic-clonic seizure with a duration of approximately 5-10 s.<br>No anaesthesia reported.<br><br>Daily for 14 days during ACTH or saline treatment.	OF,<br>FST,<br>WDS	ECS decreased the duration of the immobility time in the FST compared to sham conditions in ACTH-treated rats (n=6-8/group, p<0.05, ANOVA followed by Student's t-test). ECS increased the locomotor activity and the number of rearings (n=6-9/group, p<0.001 respectively, two-way ANOVA followed by Student's t-test) in the OF compared to sham conditions in ACTH-treated rats. ECS increased the number of DOI-induced WDS in ACTH-treated mice compared to the sham group (n=8/group, p<0.001, two-way ANOVA followed by Student's t-test). ECS increased the hippocampal BDNF protein level of ACTH-treated rats compared to sham animals (n=6-8/group, p<0.05, two-way ANOVA followed by Student's t-test). Repeated imipramine administration (10 mg/kg, daily for 14 days) had no significant effect on the hippocampal BDNF protein levels of the ACTH-treated rats (n=6-8/group, p>0,05, two-way ANOVA followed by Student's t-test).	[36]

**37**	Neuro-endocrine model<br><br>Ireland	Injection (s.c.) of corticosterone (CORT, 40 mg/kg) or saline (0.9%).<br><br>Daily for 21 days.	Sprague-Dawley<br><br>Male, (specific age not reported)	Administration of brief pulse (BP) and ultrabrief pulse (UBP) ECS via ear clip electrodes with an Ugo Basile ECT Unit 57800 device.<br>Parameter details for BP ECS: 0.5 ms width, 100 Hz frequency, 0.7 s duration and 75 mA current.<br>Parameter details for UBP ECS: 0.3 ms width, 100 Hz frequency, 0.7 s duration and 75 mA current.<br>Sham ECS was conducted procedurally similar, yet without current administration.<br>A tonic-clonic seizure was used as an ECS success marker.<br>No anaesthesia reported.<br><br>Daily for 10 days during continued corticosterone or saline treatment.	FST	BP ECS and UBP ECS reduced the immobility time in the FST compared to sham+CORT (n=14 for BP ECS+CORT vs n=13 for UBP ECS+CORT vs n=14 for sham ECS+CORT, p<0.001 and p<0.01, respectively, one-way ANOVA with Tukey's post-hoc test). There was no significant difference between BP- and UBP-treated animals (n=14 for BP ECS+CORT vs n=13 for UBP ECS+CORT, p>0.05, one-way ANOVA with Tukey's post-hoc test). The BP ECS and the UBP ECS induced an increase of hippocampal BDNF compared to sham+CORT (n=14 for BP ECS+CORT vs n=13 for UBP ECS+CORT vs n=14 for sham ECS+CORT, p<0.001 and p<0.01, respectively, one-way ANOVA with Tukey's post-hoc test). There was no significant difference between BP- and UBP-treated animals (n=14 for BP ECS+CORT vs n=13 for UBP ECS+CORT, p>0.05, one-way ANOVA with Tukey's post-hoc test). BP ECS+CORT (n=14/group, p<0.05 for frontal cortex and hippocampus, respectively, one-way ANOVA with Tukey's post-hoc test) but not UBP ECS+CORT (n=13 vs n=14 for sham ECS+CORT, p>0.05, one-way ANOVA with Tukey's post-hoc test) induced a significant increase in GFAP mRNA expression in the frontal cortex and hippocampus compared to sham ECS+CORT.	[37]
**38**	Neuro-endocrine model<br><br>Japan	Injection (s.c.) of ACTH (0.45 mg/kg) or vehicle (saline).<br><br>Daily for 18 days.	ddY<br><br>Male, 5 weeks	Administration via bilateral ear clip electrodes with an Ugo Basile ECS unit.<br>Parameter details: 30-40 mA current, 0.5 ms pulse width, 100 Hz frequency and 1 s duration.<br>Mice treated with ECS displayed a tonic-clonic seizure with a duration longer than 5 s.<br>Anaesthesia: administration of isoflurane (1.5 to 2%).	FST	An effect of ECS on immobility in the FST was not observed in the ACTH-treated mice, compared to sham animals (n=8, p>0.05, two-way ANOVA followed by Bonferroni's post-hoc test). ECS induced increased gene expression of BDNF (n=7-8, p<0.05, unpaired t-test), NPY (n=7-8, p<0,05, unpaired t-test) and Drd1 (n=7-8, p<0.01, unpaired t-test) in the hippocampus of vehicle-treated mice compared to sham. Under chronic ACTH treatment, there was no difference in the expression of these genes induced by ECS, compared to sham (n=7-8, p>0.05, unpaired t-test). Cell proliferation after ECS (n=4, for control and ACTH-treated mice: p<0.001, unpaired t-test) and the differentiation to neural progenitor cells after ECS (n=4, for control mice: p<0.001, for ACTH-treated mice: p<0.01, unpaired t-test) in the hippocampus was enhanced in ACTH-treated mice as well as in control mice compared to sham conditions.	[38]

				Daily for 11 days during continued ACTH or vehicle treatment.			
**39**	Neuro-endocrine model<br><br>France	Administration of CORT (35 µg/ml, equivalent to about 5 mg/kg/d) or vehicle (0.45% β-cyclodextrine) via the drinking water.<br><br>Daily over 10 weeks. During the last 5 weeks administration of CORT in the presence of fluoxetine (18 mg/kg/d) or vehicle.	C57BL/6JRj<br><br>Male, 7-8 weeks	Administration with an Ugo Basile ECS unit. Electrode placement not mentioned.<br>Parameter details: 50 mA current, 3 ms pulse width, 100 Hz frequency and 1 s duration.<br>Mice treated with ECS displayed a tonic-clonic seizure.<br>Anaesthesia: administration of isoflurane (2%).<br><br>Once every 2 days for a total of 7 ECS sessions over 15 days during corticosterone and fluoxetine treatment.	Elevated plus-maze (EPM),<br>NSF,<br>splash test	In the EPM, ECS in fluoxetine-non-responding (Flx-NR) mice increased time in open arms and number of entries in open arms, compared to Cort/Veh mice (n=7/group, $p<0.01$ and $p<0.001$ respectively, one-way ANOVA with post-hoc tests).<br>Time spent in open arms and number of entries in open arms in the EPM in Flx-NR mice treated with ECS were not significantly different from those measured in fluoxetine-responding (Flx-R) mice (n=7 for ECS+Flx-NR vs 18 for Flx-NR, p=0.387 and p=0.742 respectively, one-way ANOVA with post-hoc tests).<br>Non-significant decreases in the latency to feed in the NSF and non-significant increases in the grooming behaviour in the splash test were observed in the Cort/Flx-ECS group in comparison to the Cort/Veh group (n=7/group, p=0.26 and p=0.14 respectively, Kaplan–Meier survival analysis with. Mantel–Cox log rank test).<br>ECS induced significant changes in the abundance of 18 proteins in a peripheral blood mononuclear cell proteomic analysis (7 down-regulated and 11 upregulated) in Cort/Flx-NR mice. (n=3/group, $p<0.05$, Student t test).	[39]
**40**	Genetic model<br><br>Israel	Chronically elevated stress vulnerability / stress response due to selective breeding.	Sprague-Dawley (depressed vs. motivated after selective breeding)<br><br>Male, 60 days	Administration of ECS via ear clip electrodes with a Siemens Konvulsator 2077 S system.<br>Parameter details: 50 Hz frequency, 1.5 s duration, 100 V voltage.<br>Sham ECS was conducted procedurally similar, yet without current administration.<br>A tonic-clonic seizure with a duration of at least 10 s was used as an ECS success marker.<br>Anaesthesia: i.p. injection of ketamine (85 mg/kg) and promace (0.85 mg/kg)	SPT,<br>HCL,<br>FST<br><br>EPM<br>(used to test responses to breeding selection, not antidepressant efficacy).	ECS showed antidepressive effects increasing all assessed behavioural parameters (FST mobility score: p=0.0001; SPP: p=0.0001; homecage locomotion score: p=0.0337) relative to the control group (n=10-22/group, one-way ANOVA followed by Fisher's LSD test). Neither desipramine (15 mg/kg, i.p. daily for 15 days) nor fluoxetine (10 mg/kg, i.p. daily for 3 weeks) affected the behaviour of DRL rats in the FST, SPT and HCL (n=10-22/group, one-way ANOVA followed by Fisher's LSD test). ECS but not desipramine elevated BDNF protein levels ($p<0.01$) in the dorsal hippocampus of DRL rats compared to control animals (n=5-11/group, one-way ANOVA followed by Fisher's LSD test).	[40]

				Daily for 10 days.			
**41**	Genetic model<br><br>Sweden	Chronically elevated stress vulnerability / stress response due to selective breeding.	Flinders sensitive line (FSL)<br><br>Flinders resistant line (FRL)<br><br>Male, adult (specific age not reported)	Administration of ECS via ear clip electrodes with a Grass S44 stimulator.<br>Parameter details: 20 ms width, 50 Hz frequency, 1 s duration, 150 V voltage.<br>Sham ECS was conducted procedurally similar, yet without current administration.<br>All rats treated with ECS showed a tonic-clonic seizure with a duration of approximately 10-14 s.<br>No anaesthesia reported.<br><br>Every other day for a total of 8 ECS sessions.	FST	ECS induced neuropeptide Y mRNA expression in the hippocampal CA1-2 regions (n=5-6/group, p<0.0001, two-way ANOVA followed by Tukey-Kramer post-hoc and Bonferroni's correction) in the FSL compared to sham conditions. ECS increased Y_1-receptor mRNA in both FRL and FSL rats in the CA1-2 regions, in the dentate gyrus and in the parietal cortex compared to FRL and FSL sham, respectively (n=5-6/group, all p<0.0001, two-way ANOVA followed by Tukey-Kramer post-hoc and Bonferroni's correction). ECS decreased the FST immobility time in FRL and FSL rats compared to FRL sham and FSL sham, respectively (n=5/group, p<0.05 and p<0.001, respectively, two-way ANOVA followed by Bonferroni post-hoc test).	[41]
**42**	Genetic model<br><br>Israel	Chronically elevated stress vulnerability / stress response due to selective breeding.	FSL<br><br>Male, adult (specific age not reported)	Administration of ECS via ear clip electrodes with a SD9 Stimulator system.<br>Parameter details: 50 Hz frequency, 1.5 s duration, 100 V voltage.<br>Rats treated with ECS showed a tonic-clonic seizure with a duration of 10-15 s.<br>No details about the administration of sham ECS reported.<br>No anaesthesia reported.<br><br>Daily for 9 days.	FST	ECS or DHEA treatment of FSL rats decreased immobility time compared to FSL+DMSO/saline (vehicle) in the FST (n=5-14/group, p<0.05, respectively, one-way ANOVA followed by SNK-test). ECS treatment in DHEA-treated rats attenuated the antidepressive effect, increasing immobility time compared to DHEA-treated FSL rats (n=5-14/group, p<0.002, one-way ANOVA followed by a SNK-test). ECS treated rats had higher serum CORT levels than the DHEA and ECS+DHEA groups (n=7-12/group, p<0.01, respectively, one-way ANOVA followed by SNK-test). The ECS treated rats had higher cortical CORT levels than the FSL controls, the DHEA and the ECS+DHEA groups (n=8-11/group, p<0.05 for the control group and p<0.001 for DHEA and ECS+DHEA groups, respectively, one-way ANOVA followed by SNK-test).	[42]

**43**	Genetic model<br><br>Denmark	Chronically elevated stress vulnerability / stress response due to selective breeding.	FSL<br>FRL<br><br>Male, adult (specific age not reported)	Administration of ECS via ear clip electrodes with an Ugo Basile device.<br>Parameter details: square wave pulses, 55-70 mA current, 100 Hz frequency, 0.5 s duration.<br>Sham ECS was conducted procedurally similar, yet without current administration.<br>A tonic-clonic seizure with a duration less than 10 s was used as an ECS success marker. This was achieved with currents of 55 mA for FRL and 70 mA for FSL rats.<br>No anaesthesia reported.<br><br>Daily for 10 days.	FST	Following ECS, the FSL rats showed a decrease in immobility time compared with the FSL sham group (n=8/group, $p<0.001$, two-way ANOVA followed by Fisher's LSD test). The volume of the hippocampus in FSL rats was significantly increased after ECS treatment compared with the FSL sham group (n=8/group, $p<0.001$, two-way ANOVA followed by Fisher's LSD test). Following ECS, FSL rats showed a significant increase in the number of neurons in the dentate GCL when compared with the sham treated FSL group (n=8/group, $p<0.01$, two-way ANOVA followed by Fisher's LSD test). There was no significant difference in the number of neurons and glial cells in the hilus of FSL with ECS rats compared to the FSL sham group (n=8/group, $p=0.094$ and $p=0.104$, respectively, two-way ANOVA followed by Fisher's LSD test).	[43]
**44**	Genetic model<br><br>Japan	Chronically elevated stress vulnerability / stress response due to selective breeding.	Gunn rats<br>Wistar rats<br><br>Male, 8 weeks	Administration of ECS via bilateral ear clip electrodes with a E.C. MK-810 stimulator.<br>Parameter details: sine wave pulse, 50 mA current, 60 Hz frequency, 1.5 s duration.<br>A tonic-clonic seizure with a duration less than 10 s was used as an ECS success marker.<br>Sham ECS was conducted procedurally similar, yet without current administration.<br>Anaesthesia: inhalation of isoflurane (4% for initial induction) with an oxygen flow rate of 2–4L/min. During ECS continuous anaesthesia	FST,<br>YMT	ECS shortened the immobility time in the FST (n=6/group, $p<0.001$, two-way ANOVA) and improved spatial memory impairments of Gunn rats with an increase of spontaneous alternation behaviour of Gunn rats in the YMT (n=6/group, $p<0.001$, two-way ANOVA). ECS increased the astrocytic-endfoot coverage of blood vessels in the medial prefrontal cortex of Gunn rats in the prelimbic (n=5/group, $p<0.05$, two-way ANOVA), but not in the infralimbic area (n=5/group, $p>0.05$, two-way ANOVA). ECS increased the astrocytic-endfoot coverage of blood vessels in the hippocampal CA1 (n=5/group, $p<0.05$, two-way ANOVA) and CA3 (n=5/group, $p<0.05$, two-way ANOVA) region of Gunn rats, but not in the dentate gyrus (n=5/group, $p>0.05$, two-way ANOVA). ECS increased expression of aquaporin-4 (n=5/group, $p<0.05$, two-way ANOVA) and claudin-5 (n=5/group, $p<0.05$, two-way ANOVA) in the hippocampus of Gunn rats.	[44]

				with isoflurane inhalation (2%) with an oxygen flow rate of 2–4L/min.<br><br>Daily for 6 days.			
**45**	Genetic model<br><br>Canada	Chronically elevated stress vulnerability / stress response due to selective breeding.	Wistar<br>Wistar Kyoto (WKY)<br><br>Male, 7-8 weeks	Administration of ECS via ear clip electrodes. ECS device not reported.<br>Parameter details: square wave pulses, 55-65 mA amplitude, 60 Hz frequency, 0.8 s duration.<br>Sham ECS was conducted procedurally similar, yet without current administration.<br>Rats displayed tonic-clonic seizures with a duration of 45-60 s.<br>No anaesthesia reported.<br><br>Daily for 5 days.	FST,<br>OF,<br>MWM,<br>conditioned emotional response (CER)	In the FST at 1 day after the ECS treatments (post-ECS day 1), immobility time was reduced after ECS both in WKY and Wistar rats compared to respective sham controls (n=9-10/group, p<0.001 each, ANOVA). WKY treated with ECS showed a higher immobility time compared to Wistar rats treated with ECS (n=9-10/group, p<0.001, ANOVA). In the FST at post-ECS day 7, immobility time remained reduced,both in WKY and Wistar rats compared to sham controls (n=9-10/group, p<0.001 each, ANOVA). WKY treated with ECS still showed a higher immobility time compared to Wistar rats treated with ECS (n=9-10/group, p<0.001, ANOVA).<br>In the OF at post-ECS day 1, WKY (both ECS- and sham treated) demonstrated a reduced number of squares crossed compared to Wistar rats (n=9-10/group, p<0,001, ANOVA). In the OF at post-ECS day 7, the number of squares crossed was lower in ECS-treated WKY and ECS-treated Wistar rats compared to sham controls (n=9-10/group, p<0.001 each, ANOVA). Both ECS- and sham-treated WKY had a lower number of squares crossed compared to Wistar rats (n=9-10/group, all p<0.001, ANOVA).<br>In the CER at post-ECS day 1, ECS-treated WKY froze more than WKY sham (n=9-10/group, p<0.001, ANOVA). ECS-treated WKY rats froze more than ECS-treated Wistar rats (n=9-10/group, p<0.001, ANOVA). In the CER post-ECS day 7, there was no significant difference between the groups (n=9-10/group, p>0.05, ANOVA).<br>In the MWM, ECS treatment reduced time spent in the goal quadrant in the Wistar rats at both the post-ECS day 1 and 7 measurements (n=9-10/group, all p<0.001, repeated-measures ANOVA with post hoc analysis). Similarly, in the WKY rats, time spent in the goal quadrant was reduced after ECS at post-ECS days 1 and 7 (n=9-10/group, p<0.012 and p<0,02, respectively, repeated-measures ANOVA with post hoc analysis).<br>At post-ECS day 1, BDNF protein levels were elevated in ECS-treated Wistar and WKY rats compared to sham-treated Wistar and WKY rats at most brain regions investigated, including the hippocampus, frontal cortex, neocortex and brainstem (n=9-10/group, all p<0.0003, ANOVA).<br>At post ECS day 7, BDNF levels remained elevated in ECS-treated Wistar but not in WKY rats relative to respective sham controls (n=9-10/group, p<0.007 and p>0.05 respectively, ANOVA).	[45]

						At post-ECS day 1, CRF protein levels in ECS-treated WKY and Wistar rats were elevated in the hippocampus, frontal cortex, neocortex and striatum, relative to respective sham controls (n=9-10/group, p<0.0001, p<0.0001, p<0.0001 and p<0.03, respectively, ANOVA). CRF protein levels were higher in these regions in WKY rats compared to Wistar rats (n=9-10/group, p<0.0007, p<0.025, p<0.001 and p<0.0003 respectively, ANOVA). At post-ECS day 7, protein levels of CRF were still elevated in ECS- compared to respective sham-treated rats (both Wistar and WKY) in the hippocampus, frontal cortex and neocortex (n=9-10/group, p<0.0001 for all, ANOVA).	
**46**	Genetic model and CUMS<br><br>China	Stressors were applied randomly. Stressors included: cold water swimming at 4°C for 5 min; food deprivation for 24h; water deprivation for 24h; tail pinching for 1 min; social crowding with cage being tilted (30°) for 24h; shaking for 20 min (1 shake/s); continuous lighting for 24 h; housing in a soiled cage for 24h; heat stress in oven at 45°C for 5 min; undesirable confinement for 2h.<br><br>Daily for 28 days.	Wistar<br>WKY<br><br>Male, adult (specific age not reported)	Administration via ear clip electrodes with a Niviqure ECS system. Parameter details: bidirectional square wave pulses, 120 mC charge.<br>Sham ECS was conducted procedurally similar, yet without current administration. Oxygen was provided and oxygen saturation was monitored during ECS. Only rats with oxygen saturation of 95% or higher were included.<br>Anaesthesia: i.p. injection of propofol (90 mg/kg).<br><br>Daily for 7 days.	SPT,<br>OF,<br>MWM	In the SPT, the WKY+ECS group and the Wistar+CUMS+ECS group showed a higher increase in the SPP rate following ECS, compared to WKY+sham (n=12/group, p=0.001 and p<0.001 respectively, ANOVA followed by LSD test) and to Wistar+CUMS+sham (n=12/group, p=0.001 and p<0.001 respectively, ANOVA followed by LSD test). The SPP was increased following ECS in the Wistar+CUMS+ECS group compared to the WKY+ECS group (n=12/group, p<0.001, ANOVA followed by LSD test).<br>In the OF, the locomotor distance increased following ECS in the WKY+ECS group and Wistar+CUMS+ECS group compared to WKY+sham (n=8-12/group, all p<0.001, ANOVA followed by LSD test). The distance increases following ECS were higher in the WKY+ECS group and Wistar+CUMS+ECS group, compared to Wistar+CUMS+sham group (n=8-12/group, p=0.033 and p<0.001 respectively, ANOVA followed by LSD test). The distance increase after ECS was higher in the Wistar+CUMS+ECS group compared to WKY+ECS group (n=8-12/group, p<0.001, ANOVA followed by LSD test).<br>In the OF, the rearing behaviour increased after ECS in the WKY+ECS group and Wistar+CUMS+ECS group, compared to WKY+sham ECS group (n=8-12/group, p<0.001 respectively, ANOVA followed by LSD test). The rearing behaviour increased following ECS was higher in the WKY+ECS group and Wistar+CUMS+ECS group compared to Wistar+CUMS+sham (n=8-12/group, p<0.001 respectively, ANOVA followed by LSD test). An increased rearing behaviour after ECS was observed in the Wistar+CUMS+ECS group, compared to WKY+ECS group (n=8-12/group, p<0.001, ANOVA followed by LSD test).<br>In the MWM, the time in the platform quarter after ECS was smaller in the WKY+ECS group and higher in the Wistar+CUMS+ECS group, compared to WKY+sham (n=12/group, p=0.012 and p<0.001, respectively, ANOVA followed by LSD test). The platform quarter time following ECS was lower in the WKY+ECS group and higher in the Wistar+CUMS+ECS group, compared to Wistar+CUMS+sham ECS (n=12/group, p=0.002 and p<0.001 respectively, ANOVA followed by LSD test). The time in the platform quarter following ECS was higher in the	[46]

						Wistar+CUMS+ECS group, compared to the WKY+ECS group (n=12/group, p<0.001, ANOVA followed by LSD test).<br>There was no significant difference between depression model (genetic depression model, CUMS) and treatment (ECS, sham ECS) in the neuron numbers in the hippocampal CA1 or DG regions (n=6/group, all p>0.05, ANOVA followed by LSD test).<br>Hippocampal BDNF protein levels were higher in the Wistar+CUMS+ECS group compared to Wistar+CUMS+sham, WKY+sham and WKY+ECS groups (n=6/group, p<0.001 each, ANOVA followed by LSD test).<br>Hippocampal CREB protein levels were higher in the Wistar+CUMS+ECS group compared to Wistar+CUMS+sham, WKY+sham and WKY+ECS groups (n=5/group, p<0.001 each, ANOVA followed by LSD test).<br>Hippocampal p-CREB protein levels were higher in the Wistar+CUMS+ECS group compared to Wistar+CUMS+sham, WKY+sham and WKY+ECS groups (n=6/group, p=0.009, p<0.001, and p=0.038, respectively, ANOVA followed by LSD test). Hippocampal p-CREB protein levels were higher in the WKY+ECS group compared to the WKY+sham ECS group (n=6/group, p=0.013, ANOVA followed by LSD test).	
**47**	Genetic and neuro-endocrine model<br><br>France	Chronically elevated stress vulnerability / stress response due to selective breeding.<br><br>Administration of corticosterone (CORT, 35 µg/ml/d) via the drinking water.<br>Daily administration for 6 weeks prior to ECS and continued during and after ECS.	Microtubule-associated protein 6 knock out mice C57BL/6J<br><br>Male, 2-5 months	Administration via bilateral ear clip electrodes with an Ugo Basile ECS unit.<br>Parameter details: 20 mA current, 0.5 ms pulse width, 120 Hz frequency and 2 s duration. Microtubule-associated protein 6 knock-out mice were used to estimate seizure threshold in ECS.<br>Sham ECS was conducted procedurally similar, yet without current administration.<br>Anaesthesia: administration of isoflurane, no further details reported.<br><br>Initial sessions: 10 ECS sessions over a period of 12 days.	FST, NSF	ECS resulted in decreased immobility (n=11 for ECS vs 10 for sham, p=0.0138, Mann-Whitney test) and increased climbing (n=11 for ECS vs 10 for sham, p=0.0166, Mann-Whitney test) activity in the FST in knock out animals compared to sham. ECS decreased the latency to eat in the NSF in knockout animals compared to sham (n=13/group, p=0.0289, Mann-Whitney test).<br>ECS treatment increased the number of BrdU-positive cells in the sub-granular zone of the hippocampus (n=5 for ECS vs 4 for sham, p=0.0278, Mann-Whitney test), of DCX-positive cells in the granular cell layer of the hippocampus (n=8/group, p<0.0001, Mann-Whitney test), and of hippocampal BDNF mRNA levels (n=7/group, p=0.0087, Mann-Whitney test) and BDNF protein levels (n=10 for ECS vs 12 for sham, p<0.0001, Mann-Whitney test) in knock out mice compared to sham. ECS treatment increased the number of BrdU-positive cells in the subventricular zone of the hippocampus in knock out animals (n=8/group, p=0.0076, Mann-Whitney test).<br>ECS treatment increased the number of EdU-positive cells in the subventricular zone of the hippocampus (n=10/group, p<0.0001, Mann-Whitney test) in CORT mice compared to sham.<br>ECS increased cortical neuronal spine density in knock out animals compared to sham (n=26 dendritic segments from 3 ECS mice vs 11 dendritic segments from 3 sham mice, p=0.0303, Mann-Whitney test). Significant NSF effects in knock out animals were described up until day 17 following ECS (n=7/group, p=0.0055, Mann-Whitney test), while a significant increase in EdU-positive cells in the hippocampus of knock out animals was found up until day 40 following ECS (n=6 for ECS vs 5 for sham,	[47]

				Continuation sessions: 2 ECS sessions per week for 5 weeks.		p=0.0273, Mann-Whitney test), in addition to a negative correlation (n=19/group, p=0.0077) between the latency to eat and the amount of EdU-positive hippocampal cells. Knock out animals with ECS continuation treatment displayed a reduced latency to eat (n=9 for ECS/ECS vs 10 for ECS/sham, p=0.011, Mann-Whitney test) in the NSF compared to animals with only the initial ECS treatment and an increased number of BrdU-positive cells in the hippocampus (n=9/group, p=0.0252, Mann-Whitney test).	
**48**	Genetic and neuro-endocrine model<br><br>USA	Administration of CORT (35µg/ml/d) or vehicle (0.45% hydroxypropyl-β-cyclodextrine) via the drinking water.<br><br>Daily for 6 weeks.	hGFAPtk mice (animals with a suppression of neurogenesis in actively dividing GFAP-expressing cells in adulthood) and wild type C57BL/6J<br><br>Male, 8 weeks	Administration with an Ugo Basile ECS unit. Electrode placement not reported. Parameter details: 50 mA current, 3 ms pulse width, 100 Hz frequency and 1 s duration. No details about sham ECS reported. Mice treated with ECS displayed a tonic-clonic seizure. Anaesthesia: administration of isoflurane, no further details reported.<br><br>7 ECS sessions over 15 days during CORT or vehicle treatment.	NSF, grooming test, investigation of animal's coat state	ECS reversed the effects of CORT administration in C57BL/6J mice by reducing the latency to eat in the NSF (n=12 for ECS vs 11 for sham, p<0.0001, two-way ANOVA with Student-Newman-Keuls (SNK) test), reducing the latency to groom in the grooming test (n=12 for ECS vs 11 for sham, p<0.0001, two-way ANOVA with SNK test) and reducing the coat state scores (n=12 for ECS vs 11 for sham, p<0.0001, two-way ANOVA with SNK test) compared to CORT+sham. ECS increased the number of DCX positive cells in the hippocampus (n=6 for ECS vs 5 for sham, p=0.0005, two-way ANOVA with Bonferroni-Dunn post-hoc test). There was no significant difference in latency to eat in the NSF, in latency to groom in the grooming test and in the coat state score between ECS and sham in hGFAPtk+VGCV mice without neurogenesis (n=9 for ECS vs 8 for sham, all p>0.05, two-way ANOVA with SNK test)	[48]

References

1. Ren, L., et al., *Propofol ameliorates electroconvulsive shock-induced learning and memory impairment by regulation of synaptic metaplasticity via autophosphorylation of CaMKIIa at Thr 305 in stressed rats.* Psychiatry Res, 2016. **240**: p. 123-130.
2. Wu, B., et al., *Reduced Synaptic Plasticity Contributes to Resistance Against Constant-Stimulus Electroconvulsive Treatment in a Rat Model of Stress-Induced Depression.* Neuropsychiatr Dis Treat, 2021. **17**: p. 1433-1442.
3. Zhang, F., et al., *Propofol alleviates electroconvulsive shock-induced memory impairment by modulating proBDNF/mBDNF ratio in depressive rats.* Brain Res, 2016. **1642**: p. 43-50.
4. Zhang, Y., et al., *Effects of N-Methyl-D-aspartate receptor (NMDAR) and Ca(2+)/calmodulin-dependent protein kinase IIalpha (CaMKIIalpha) on learning and memory impairment in depressed rats with different charge by modified electroconvulsive shock.* Ann Transl Med, 2021. **9**(16): p. 1320.
5. Zhu, X., et al., *Propofol inhibits inflammatory cytokine-mediated glutamate uptake dysfunction to alleviate learning/memory impairment in depressed rats undergoing electroconvulsive shock.* Brain Res, 2015. **1595**: p. 101-9.
6. Zhu, X., et al., *Ketamine-mediated alleviation of electroconvulsive shock-induced memory impairment is associated with the regulation of neuroinflammation and soluble amyloid-beta peptide in depressive-like rats.* Neurosci Lett, 2015. **599**: p. 32-7.
7. Ren, L., et al., *Anesthetics alleviate learning and memory impairment induced by electroconvulsive shock by regulation of NMDA receptor-mediated metaplasticity in depressive rats.* Neurobiol Learn Mem, 2018. **155**: p. 65-77.
8. Chen, J., et al., *Effects of low-dose ketamine combined with propofol on phosphorylation of AMPA receptor GluR1 subunit and GABAA receptor in hippocampus of stressed rats receiving electroconvulsive shock.* J ECT, 2015. **31**(1): p. 50-6.
9. Lv, F., et al., *Effects of propofol on expression of hippocampal neuronal nitric oxide synthase and carboxy-terminal PDZ ligand of neuronal nitric oxide synthase in stressed rats undergoing electroconvulsive shock.* J ECT, 2013. **29**(4): p. 297-302.
10. Luo, J., et al., *Propofol protects against impairment of learning-memory and imbalance of hippocampal Glu/GABA induced by electroconvulsive shock in depressed rats.* J Anesth, 2011. **25**(5): p. 657-65.
11. Gao, X., et al., *Dexmedetomidine protects against learning and memory impairments caused by electroconvulsive shock in depressed rats: Involvement of the NMDA receptor subunit 2B (NR2B)-ERK signaling pathway.* Psychiatry Res, 2016. **243**: p. 446-52.
12. Dong, J., et al., *Effects of electroconvulsive therapy and propofol on spatial memory and glutamatergic system in hippocampus of depressed rats.* J ECT, 2010. **26**(2): p. 126-30.

13. Yu, J., et al., *Inhibition of CB1 receptor alleviates electroconvulsive shock-induced memory impairment by regulating hippocampal synaptic plasticity in depressive rats.* Psychiatry Res, 2021. **300**: p. 113917.
14. Li, W., et al., *Effects of electroconvulsive stimulation on long-term potentiation and synaptophysin in the hippocampus of rats with depressive behavior.* J ect, 2012. **28**(2): p. 111-7.
15. Zhong, X., et al., *(2R,6R)-Hydroxynorketamine Alleviates Electroconvulsive Shock-Induced Learning Impairment by Inhibiting Autophagy.* Neuropsychiatr Dis Treat, 2021. **17**: p. 297-304.
16. Chen, L., et al., *Roles of prokineticin 2 in electroconvulsive shock-induced memory impairment via regulation of phenotype polarization in astrocytes.* Behav Brain Res, 2023. **446**: p. 114350.
17. Li, X., et al., *Effects of propofol on the activation of hippocampal CaMKIIalpha in depressed rats receiving electroconvulsive therapy.* J ECT, 2012. **28**(4): p. 242-7.
18. Gersner, R., et al., *Site-specific antidepressant effects of repeated subconvulsive electrical stimulation: potential role of brain-derived neurotrophic factor.* Biol Psychiatry, 2010. **67**(2): p. 125-32.
19. Zhang, F., G. Huang, and X. Zhu, *Effect of different charges of modified electroconvulsive seizure on the cognitive behavior in stressed rats: Effects of GluR1 phosphorylation and CaMKIIalpha activity.* Exp Ther Med, 2019. **17**(1): p. 748-758.
20. Luo, J., et al., *Propofol interacts with stimulus intensities of electroconvulsive shock to regulate behavior and hippocampal BDNF in a rat model of depression.* Psychiatry Res, 2012. **198**(2): p. 300-6.
21. Luo, J., et al., *Propofol prevents electroconvulsive-shock-induced memory impairment through regulation of hippocampal synaptic plasticity in a rat model of depression.* Neuropsychiatr Dis Treat, 2014. **10**: p. 1847-59.
22. Neyazi, A., et al., *P11 promoter methylation predicts the antidepressant effect of electroconvulsive therapy.* Transl Psychiatry, 2018. **8**(1): p. 25.
23. Chen, Q., et al., *Changes in synaptic plasticity are associated with electroconvulsive shock-induced learning and memory impairment in rats with depression-like behavior.* Neuropsychiatr Dis Treat, 2018. **14**: p. 1737-1746.
24. Li, P., et al., *Propofol Mitigates Learning and Memory Impairment After Electroconvulsive Shock in Depressed Rats by Inhibiting Autophagy in the Hippocampus.* Med Sci Monit, 2016. **22**: p. 1702-8.
25. Liu, D., et al., *Inhibition of NLRP3 inflammasome-mediated neuroinflammation alleviates electroconvulsive shock-induced memory impairment via regulation of hippocampal synaptic plasticity in depressive rats.* Behav Brain Res, 2022. **428**: p. 113879.

26. Rimmerman, N., et al., *Microglia and their LAG3 checkpoint underlie the antidepressant and neurogenesis-enhancing effects of electroconvulsive stimulation.* Mol Psychiatry, 2022. **27**(2): p. 1120-1135.
27. Olesen, M.V., et al., *Electroconvulsive stimulation results in long-term survival of newly generated hippocampal neurons in rats.* Hippocampus, 2017. **27**(1): p. 52-60.
28. Olesen, M.V., G. Wörtwein, and B. Pakkenberg, *Electroconvulsive stimulation, but not chronic restraint stress, causes structural alterations in adult rat hippocampus--a stereological study.* Hippocampus, 2015. **25**(1): p. 72-80.
29. Hageman, I., et al., *Electroconvulsive stimulations normalizes stress-induced changes in the glucocorticoid receptor and behaviour.* Behav Brain Res, 2009. **196**(1): p. 71-7.
30. Nakamura-Maruyama, E., et al., *Ryanodine receptors are involved in the improvement of depression-like behaviors through electroconvulsive shock in stressed mice.* Brain Stimul, 2021. **14**(1): p. 36-47.
31. van Buel, E.M., et al., *Mouse repeated electroconvulsive seizure (ECS) does not reverse social stress effects but does induce behavioral and hippocampal changes relevant to electroconvulsive therapy (ECT) side-effects in the treatment of depression.* PLoS One, 2017. **12**(9): p. e0184603.
32. Abelaira, H.M., et al., *Combination of electroconvulsive stimulation with ketamine or escitalopram protects the brain against inflammation and oxidative stress induced by maternal deprivation and is critical for associated behaviors in male and female rats.* Mol Neurobiol, 2022. **59**(3): p. 1452-1475.
33. Liu, G., C. Liu, and X.N. Zhang, *Comparison of the neuropsychological mechanisms of 2,6-diisopropylphenol and N-methyl-D-aspartate receptor antagonist against electroconvulsive therapy-induced learning and memory impairment in depressed rats.* Mol Med Rep, 2015. **12**(3): p. 3297-3308.
34. Alizadeh Makvandi, A., et al., *Hesperetin ameliorates electroconvulsive therapy-induced memory impairment through regulation of hippocampal BDNF and oxidative stress in a rat model of depression.* J Chem Neuroanat, 2021. **117**: p. 102001.
35. Kozuru, T., et al., *Chronic electroconvulsive shock decreases (+/-) 1-(4-iodo-2,5-dimethoxyphenyl)-2-aminopropane hydrochloride (DOI)-induced wet-dog shake behaviors of dexamethasone-treated rats.* Life Sci, 2000. **66**(13): p. 1271-9.
36. Li, B., et al., *Repeated electroconvulsive stimuli increase brain-derived neurotrophic factor in ACTH-treated rats.* Eur J Pharmacol, 2006. **529**(1-3): p. 114-21.
37. O'Donovan, S., et al., *Effects of brief pulse and ultrabrief pulse electroconvulsive stimulation on rodent brain and behaviour in the corticosterone model of depression.* Int J Neuropsychopharmacol, 2014. **17**(9): p. 1477-86.

38. Kobayashi, Y. and E. Segi-Nishida, *Search for factors contributing to resistance to the electroconvulsive seizure treatment model using adrenocorticotrophic hormone-treated mice.* Pharmacol Biochem Behav, 2019. **186**: p. 172767.
39. Lebeau, R.H., et al., *Peripheral proteomic changes after electroconvulsive seizures in a rodent model of non-response to chronic fluoxetine.* Front Pharmacol, 2022. **13**: p. 993449.
40. Gersner, R., et al., *Inherited behaviors, BDNF expression and response to treatment in a novel multifactorial rat model for depression.* Int J Neuropsychopharmacol, 2014. **17**(6): p. 945-55.
41. Jimenez-Vasquez, P.A., et al., *Electroconvulsive stimuli selectively affect behavior and neuropeptide Y (NPY) and NPY Y(1) receptor gene expressions in hippocampus and hypothalamus of Flinders Sensitive Line rat model of depression.* Eur Neuropsychopharmacol, 2007. **17**(4): p. 298-308.
42. Maayan, R., et al., *The involvement of dehydroepiandrosterone (DHEA) and its sulfate ester (DHEAS) in blocking the therapeutic effect of electroconvulsive shocks in an animal model of depression.* Eur Neuropsychopharmacol, 2005. **15**(3): p. 253-62.
43. Kaae, S.S., et al., *Quantitative hippocampal structural changes following electroconvulsive seizure treatment in a rat model of depression.* Synapse, 2012. **66**(8): p. 667-76.
44. Azis, I.A., et al., *Electroconvulsive shock restores the decreased coverage of brain blood vessels by astrocytic endfeet and ameliorates depressive-like behavior.* J Affect Disord, 2019. **257**: p. 331-339.
45. Kyeremanteng, C., et al., *Effects of electroconvulsive seizures on depression-related behavior, memory and neurochemical changes in Wistar and Wistar-Kyoto rats.* Prog Neuropsychopharmacol Biol Psychiatry, 2014. **54**: p. 170-8.
46. Luo, J., et al., *Behavioral and molecular responses to electroconvulsive shock differ between genetic and environmental rat models of depression.* Psychiatry Res, 2015. **226**(2-3): p. 451-60.
47. Jonckheere, J., et al., *Short- and long-term efficacy of electroconvulsive stimulation in animal models of depression: The essential role of neuronal survival.* Brain Stimul, 2018. **11**(6): p. 1336-1347.
48. Schloesser, R.J., et al., *Antidepressant-like Effects of Electroconvulsive Seizures Require Adult Neurogenesis in a Neuroendocrine Model of Depression.* Brain Stimul, 2015. **8**(5): p. 862-7.
