# Supplementary material for: Contemporary in vivo rodent electroconvulsive therapy (ECT) models in translational depression research: a systematic review
Source: Transl Psychiatry. 2025 Nov 29;15:515. doi: 10.1038/s41398-025-03749-x (PMC12669789; doi:10.1038/s41398-025-03749-x)
Supplement: Supplementary file 2 — Supplementary Table 2: Distribution of ECS effects per behavioral test and study, subdivided into beneficial, no, or adverse impact on animal phenotypes. [file 41398_2025_3749_MOESM2_ESM.pdf]

Supplementary Table 2: Distribution of ECS effects per behavioral test and study, subdivided into beneficial, no, or adverse impact on animal phenotypes.

Test	ECS improved stress-induced deficits	ECS had no effect on stress-induced deficits	ECS aggravated stress-induced deficits
**SPT**	Ren_2016		
	Ren_2018		
	Chen_2015		
	Lv_2013		
	Luo_2011		
	Gao_2016		
	Yu_2021		
	W.Li_2012		
	Chen_2023		
	Xi.Li_2012		
	Gersner_2010		
	Zhang_2019		
	Luo_2012		
	Luo_2014		
	Neyazi_2018		
	Alizadeh_2021		
	Gersner_2014		
	Wu_2021		
	Zhang_2016		
	Zhang_2021		
	Zhu_Hao_2015		
	Zhu_Li_2015		
	Rimmerman_2022		
	Chen_2018		
	Li_2016		
	Liu_2022		
	Luo_2015		
**Total**	27	0	0

Test	ECS improved stress-induced deficits	ECS had no effect on stress-induced deficits	ECS aggravated stress-induced deficits
**Splash test**	Abelaira_2022 (male rats)	Lebeau_2022	
	Schloesser_2015	Abelaira_2022 (female rats)	

**Total**	2	2	0

Test	ECS improved stress-induced deficits	ECS had no effect on stress-induced deficits	ECS aggravated stress-induced deficits
**OFT**	Chen_2015	Abelaira_2022	Kyeremanteng_2014
	Lv_2013		
	Luo_2011		
	Dong_2010		
	Yu_2021		
	W.Li_2012		
	Chen_2023		
	Xi.Li_2012		
	Zhang_2019		
	Luo_2012		
	Luo_2014		
	Li_2006		
	Zhang_2021		
	Li_2016		
	Liu_2022		
	Luo_2015		
**Total**	16	1	1

Test	ECS improved stress-induced deficits	ECS had no effect on stress-induced deficits	ECS aggravated stress-induced deficits
**Home cage locomotion/locom**	Gersner_2014	Gersner_2010	
	Nakamura_2021		

**Total**	2	1	0

Test	ECS improved stress-induced deficits	ECS had no effect on stress-induced deficits	ECS aggravated stress-induced deficits
**FST**	Chen_2023	Gersner_2010	
	Neyazi_2018	Alizadeh_2021	
	Olesen_2017	Kobayashi_2019	
	Li_2006	Abelaira_2022 (female rats)	
	O'Donovan_2014		
	Jimenez_2007		
	Maayan_2005		
	Kaae_2012		
	Gersner_2014		
	Olesen_2015		
	Nakamura_2021		
	Abelaira_2022 (male rats)		
	Rimmerman_2022		
	Jonckheere_2018		
	Azis _2019		
	Liu_2022		
	Kyeremanteng_2014		
	Hagemann_2009		
**Total**	18	4	0

Test	ECS improved stress-induced deficits	ECS had no effect on stress-induced deficits	ECS aggravated stress-induced deficits
**MWM**	Zhang_2019		Ren_2016
	Luo_2015 (Wistar+CUMS)		Ren_2018
			Chen_2015
			Lv_2013
			Luo_2011
			Gao_2016
			Dong_2010
			Yu_2021
			Zhong_2021
			Chen_2023
			Xi.Li_2012
			Gersner_2010
			Luo_2014
			Liu_2015
			Wu_2021
			Zhang_2016
			Zhang_2021
			Zhu_Hao_2015
			Zhu_Li_2015
			Chen_2018
			Li_2016
			Liu_2022
			Kyeremanteng_2014
			Luo_2015 (WKY)
**Total**	2	0	24

Test	ECS improved stress-induced deficits	ECS had no effect on stress-induced deficits	ECS aggravated stress-induced deficits
**Y maze**	Azis_2019		Alizadeh_2021

**Total**	1	0	1

Test	ECS improved stress-induced deficits	ECS had no effect on stress-induced deficits	ECS aggravated stress-induced deficits
**NORT**			Alizadeh_2021

**Total**	0	0	1

Test	ECS improved stress-induced deficits	ECS had no effect on stress-induced deficits	ECS aggravated stress-induced deficits
**EPM**	Lebeau_2022		
**Total**	1	0	0

Test	ECS improved stress-induced deficits	ECS had no effect on stress-induced deficits	ECS aggravated stress-induced deficits
**Novelty-induced hypophagia test/NSF**	Nakamura_2021	Neyazi_2018	
	Schloesser_2015	Lebeau_2022	
	Jonckheere_2018		

**Total**	3	2	0

Test	ECS improved stress-induced deficits	ECS had no effect on stress-induced deficits	ECS aggravated stress-induced deficits
**WDS**	Li_2006		Kozuru_2000

**Total**	1	0	1

Test	ECS improved stress-induced deficits	ECS had no effect on stress-induced deficits	ECS aggravated stress-induced deficits
**Social interaction/ exploration test**		Neyazi_2018	
		Rimmerman_2022	

**Total**	0	2	0

Test	ECS improved stress-induced deficits	ECS had no effect on stress-induced deficits	ECS aggravated stress-induced deficits
**Exploration and novelty-induced behavior**		Gersner_2010	

Total 0 1 0
